# DFT and IsoStar Analyses to Assess the Utility of σ‐ and π‐Hole Interactions for Crystal Engineering

**DOI:** 10.1002/cphc.202000927

**Published:** 2020-12-22

**Authors:** Tiddo Jonathan Mooibroek

**Affiliations:** ^1^ van ‘t Hoff Institute for Molecular Sciences Universiteit van Amsterdam, Science Park 904 1098 XH Amsterdam The Netherlands

**Keywords:** crystal engineering, density functional calculations, molecular recognition, noncovalent interactions, supramolecular chemistry

## Abstract

The interpretation of 36 charge neutral ‘contact pairs’ from the IsoStar database was supported by DFT calculations of model molecules **1**–**12**, and bimolecular adducts thereof. The ‘central groups’ are σ‐hole donors (H_2_O and aromatic C−I), π‐hole donors (R−C(O)Me, R−NO_2_ and R−C_6_F_5_) and for comparison R−C_6_H_5_ (R=any group or atom). The ‘contact groups’ are hydrogen bond donors X−H (X=N, O, S, or R_2_C, or R_3_C) and lone‐pair containing fragments (R_3_C−F, R−C≡N and R_2_C=O). Nearly all the IsoStar distributions follow expectations based on the electrostatic potential of the ‘central‐’ and ‘contact group’. Interaction energies (ΔE^BSSE^) are dominated by electrostatics (particularly between two polarized molecules) or dispersion (especially in case of large contact area). Orbital interactions never dominate, but could be significant (∼30 %) and of the *n*/π→σ*/π* kind. The largest degree of directionality in the IsoStar plots was typically observed for adducts more stable than ΔE^BSSE^≈−4 kcal⋅mol^−1^, which can be seen as a benchmark‐value for the utility of an interaction in crystal engineering. This benchmark could be met with all the σ‐ and π‐hole donors studied.

## Graphical abstract

**DFT and IsoStar analyses** are performed to assess the utility of σ‐ and π‐hole interactions for crystal engineering. The IsoStar distributions largely follow expectations based on the electrostatic potential of molecules that model the interacting molecular fragments. The directionality in the IsoStar plots was typically largest for bimolecular adducts of the model molecules more stable than ΔE^BSSE^≈−4 kcal mol^−1^. This value can be seen as a benchmark for the utility of an interaction in crystal engineering.

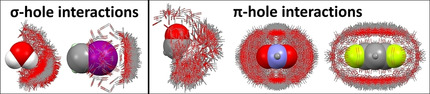

## 1 Introduction

Important phenomena such as protein folding, molecular recognition aggregation and crystallization are driven largely by non‐covalent interactions.^[1]^ The intermolecular interactions that are best known are the hydrogen^[2]^ and halogen bond.[^1a^, ^3^] Both can be seen as example of a ‘σ‐hole interaction’, where the term ‘σ ‐hole’ refers to a feature of the electron density which often results in a positive electrostatic potential on the extension (or nearly so) of the bond to that particular atom.^[4]^ The location of this Lewis acidic site coincides with the σ* orbital of that bond. The conclusion of a σ‐hole interaction can be the breaking and/or making of a σ bond. For example in acid‐base reactions^[5]^ or in the formation of [I_3_]^−^ following I^−^ attack on molecular iodine.[^5^, ^6^] Provided with the appropriate chemical framework, any non‐metal of the periodic table could in principle be rendered a σ‐hole.^[7]^ For example, Chalcogen bonding interactions have been defined by the IUPAC^[8]^ and exploited experimentally in dithienothiophenes^[9]^ as catalysts,^[10]^ transmembrane anion transporters^[11]^ and mechanosensitive fluorescent probes.[^9^, ^12^] In a very similar fashion to the σ‐hole concept, one can view electron deficient π‐systems as π‐holes[^1b^, ^13^] that can interact favorably with electron‐rich entities like lone‐pair or π‐electrons.^[14]^ Such interactions are known to occur with carbonyls,^[15]^ nitro‐compounds^[16]^ and π‐acidic aromatics.^[17]^

There are many computational studies that highlight the stabilizing effect of such σ‐ and π‐hole interactions on gas‐phase molecular adducts or crystal structures.[^7^, ^18^] However, little is known about the actual utility of novel σ‐ and π‐hole interactions as supramolecular synthons^[19]^ for (molecular recognition in) crystal engineering.^[20]^ A conventional manner of addressing this issue is to conduct thorough surveys of the Cambridge Structure Database (CSD),^[21]^ which contains over a million accurate three‐dimensional coordinates of a large range of molecules. The implicit assumption of such enquiries is that specific non‐covalent interactions can program the manner in which molecules pack with themselves, or that they can determine the relative orientation of molecules in co‐crystals such as in host‐guest complexes.

Approaches to analyse geometric data in the CSD range from simple two‐dimensional plots correlating geometric and/or numeric data to more realistic three‐dimensional (density) plots. IsoStar is a particularly intuitive software tool that has been available since around 2000.^[22]^ With IsoStar, it is possible to visualize structure overlays of parts of individual crystal structures, where the ‘central groups’ (e. g. nitro groups ‘C−NO_2_’) have been aligned, and are surrounded by the so‐called ‘contact groups’ (e. g. carbonyl groups ‘R_2_C=O’). The result is a three dimensional distribution of contact groups around the (average) central group and gives an intuitive visual representation of the directionality of a certain combination of interacting moieties. The tool has been applied broadly to the CSD leading to the online IsoStar database (currently version 2.3.4.),^[23]^ consisting of a combination of about 300 ‘central groups’ with about 50 ‘contact groups’.

The utilization of the IsoStar database in the scientific literature is surprisingly rare, presumably due the subscription‐based access of the database. In the original 1997 paper, the program was used to visualize geometric preferences of certain hydrogen bonding and π‐π stacking interactions.^[22]^ IsoStar has subsequently been used to study hydrogen bonding to lone‐pair[^21b^, ^24^] and π‐electrons,^[25]^ hydrophobic interactions,^[25a]^ halogen bonding,^[24b]^ anion‐π interactions,^[14c]^ S⋅⋅⋅N interactions,^[26]^ and interactions involving nitriles.^[27]^ These studies notwithstanding, a comprehensive and comparative evaluation of different σ‐ and π‐hole interactions present in the IsoStar database is currently unavailable. Such an evaluation might be helpful to assess the actual utility of certain σ‐ or π‐hole interaction. Herein, such a study is provided by scrutinizing about 40 carefully selected IsoStar database entries, supplemented by DFT calculations of about 70 bimolecular adducts.

## 2 Methods and Resources

### 2.1 General Considerations for the IsoStar Database Analysis

The default setting of the IsoStar database is to return structure information from the CSD when the interatomic distance between any atom of the central group and any atom of the contact group is within the van der Waals radii of the elements involved (according to Bondi),^[28]^ plus a tolerance of 0.5 Å. The resulting plots can be very congested and the graphical representations shown in this paper are limited to the entries displaying van der Waals overlap (a build‐in feature of IsoStar). The density of a certain atom of the contact groups can be visualised by a contoured density surface that has been color coded in decreasing density as: red>green>blue. Such plots are always provided as well.

At date of writing, the IsoStar database contains 279 central groups, devided in 76 termial groups, 58 acyclic links, 130 ring systems and 15 solvent molecules. Each central group has (when possibe) been combined with 47 contact groups, leading to a potential combined total of about 13,000 individual IsoStar plots. Not all of these possible combinations are present (or numerous) in the CSD, while other combinations must be very numerous (e. g. ROH with polar H's). The IsoStar files available online contain a maximum of about 5000 individual structures. In cases where more structures are actually present in the CSD, the software used to generate the IsoStar plots (IsoGen) randomly omits structures untill ≤5000 structures remain. An implicit assumption of this apprach is that pruning the data in this manner does not lead to loss of information regarding the directionality of an interaction pair.

## 3 Overview of Selected IsoStar ‘Central Groups’ and ‘Contact Goups’

A numerical overview of the amount of data found in the IsoStar database for each pair of central and contact groups is given in Table 1. From the amount of data for each contact pair it can be seen that several combinations must represent the entire dataset present in the CSD (<5000), while many others are likely compressed to contain about 5000 contact pairs.

**Table 1:** Numerical overview of the amount of hits listed in the IsoStar database version 2.3 (consulted on the 9^th^ of December 2019). X=O, N or S. R can be any atom or group. All groups are charge‐neutral.

		Central group in IsoStar					
		σ‐hole interaction		π‐hole interaction			
		H_2_O	^Ar^C‐I	RC(O)Me	RNO_2_	RC_6_F_5_	RC_6_H_5_
Contact group in IsoStar	X−H	4,986	1,626	1,968	4,993	1,220	4,973
	arom. C−H	4,946	4,955	1,958	4,983	4,992	4,993
	alkyl C−H	4,998	4,902	1,967	4,996	4,993	4,993
	C−F	2,436	2,246	1,981	2,264	4,993	4,957
	RC≡N	1,515	222	736	1,571	442	3,392
	R_2_C=O	4,998	1,268	1,998	4,993	1,092	4,993
	phenyl	1,427	1,076	970	1,412	1,426	2,496

As σ‐hole donors and **‘central groups’**, water solvate molecules (H_2_O) were used as a prototypical hydrogen bond donor and aromatic C−I moieties (^Ar^C−I) as typical halogen bond donors. The π‐hole donors selected as central group were termial acetyls (RC(O)Me, R=any group or atom), any nitro group (RNO_2_), and pentafluorophenyl rings (RC_6_F_5_). For comparison purposes, a phenyl ring (RC_6_H_5_) was also used. As interaction ‘**contact groups**’, three moieties were chosen to represent a hydrogen bonding interaction: any polar X–H fragment (X=N, O, S), any aromatic C−H, and any alkyl C−H. As electron rich partners, it was chosen to use any C−F, any nitrile (RC≡N), any carbonyl (R_2_C=O) and the centre of a phenyl ring (i. e. π‐electrons).

## 4 Calculations

**Figure 1:**
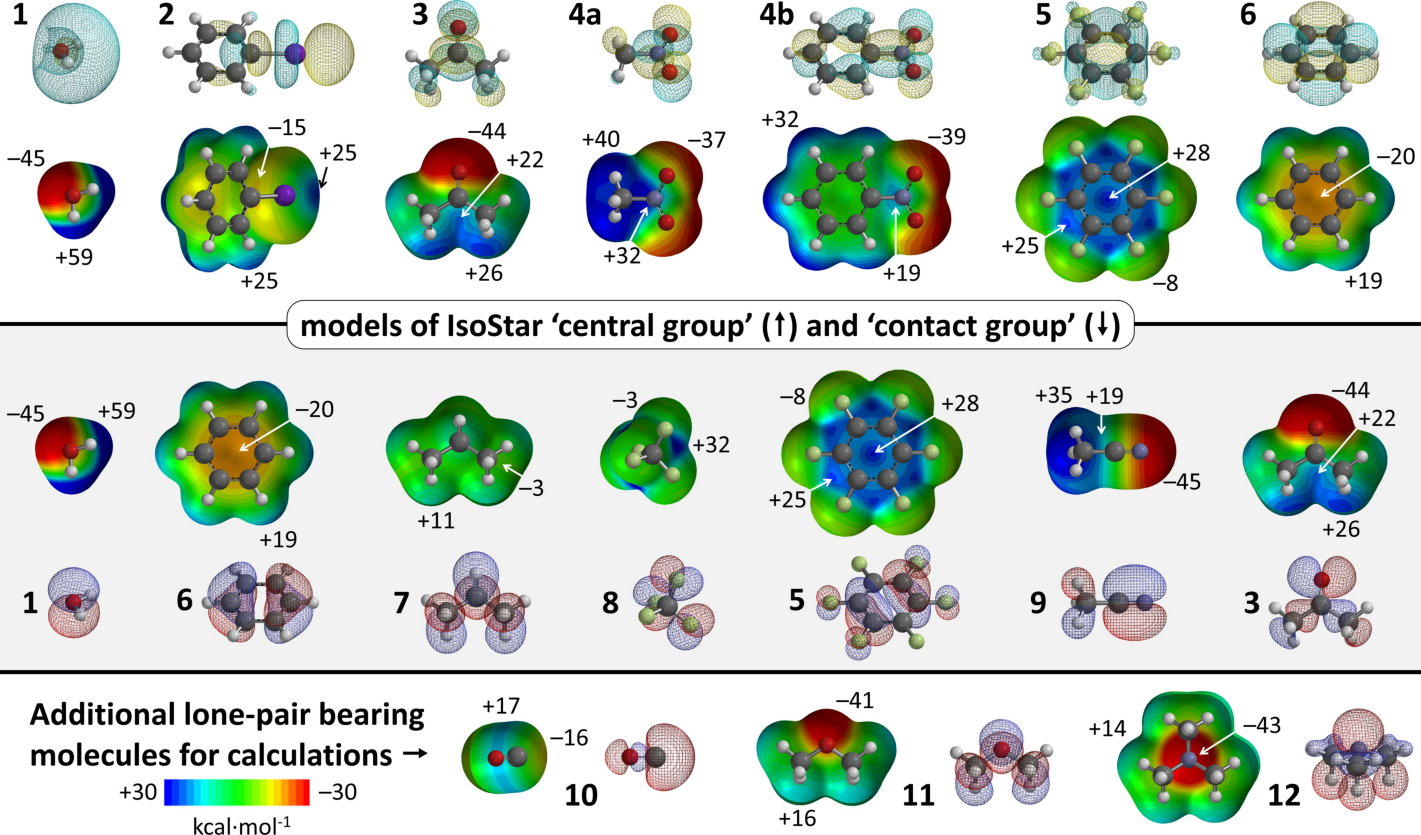
Molecules **1**–**6** (top) representing the central groups used in the IsoStar analysis, while molecules **1**, **3** and **6**–**9** (middle) represent the contact groups used in the IsoStar analysis. Molecules **10**–**12** (bottom) are additional lone‐pair bearing molecules used for computational evaluations involving a lone‐pair on C (**10**), O (**11**) and N (**12**). The LUMOs for **1**–**6**, the HOMOs for **1**, **3**, **6**–**12** and MEP's of **1**–**12** were computed using DFT at the ωB96X−D/6–31+G** level of theory. See Figure S1 for all the HOMOs and LUMOs.

Geometry optimizations were performed using Density Functional Theory (DFT) calculations with Spartan 2016 at the B3LYP^[29]^‐D3^[30]^/def2‐TZVP^[31]^ level of theory, which is known to give accurate results at reasonable computational cost and a very low basis set superposition error (BSSE).[^30^, ^31^] The molecular Electrostatic Potential maps (MEPs) and the HOMO/LUMOs displayed in Figure 1 were calculated at the ωB96X−D/6–31+G** level of theory. Molecular fragments of adducts were manually oriented in a suitable constellation before starting an unconstrained geometry optimization. The Amsterdam Density Functional (ADF)^[32]^ modelling suite at the B3LYP^[29]^‐D3^[30]^/TZ2P^[31]^ level of theory (no frozen cores) was used to compute the reported energies (ΔE^BSSE^, using the ‘ghost atoms’ option for counterpoise correction of the basis set superposition error) of the optimized structures, as well as the adducts observed in the crystal structures. ADF was likewise used to compute the energy decomposition and ‘atoms in molecules’^[33]^ analyses (using the default ADF settings, which are based on ΔE, not ΔE^BSSE^). Details of the Morokuma‐Ziegler inspired energy decomposition scheme used in the ADF‐suite have been reported elsewhere[^32^, ^34^] and the scheme has proven useful to evaluate hydrogen bonding interactions.^[35]^ The analysis of orbital interactions was conducted with ADF and visualized with the ‘view levels’ option.

CrystalExplorer 17.5 (https://crystalexplorer.scb.uwa.edu.au) was used to compute Hirshfeld surfaces and estimate interaction energies in the crystal structures (see Figure 8).^[36]^

## 5 Results and Discussion

### 5.1 Molecular Electrostatic Potential Maps and Frontier Molecular Orbitals of Models 1–12

Molecules representing the ‘central groups’ of the IsoStar analysis are modelled by molecules **1**–**6** shown in the top of Figure 1; i. e.: water (**1**), aromatic iodide **2**, carbonyl **3**, nitros **4**, π‐acidic aromatic **5**, and π‐basic aromatic **6**. Central groups **1–5** are anticiated to act primarily as σ‐hole (**1**, **2**) or π‐hole (**3**–**5**), while benzene (**6**) was used for comparison purposes (i. e., the inverse electrostatic potential of **5**).^[37]^ Models of the ‘contact groups’ (middle in Figure 1) are represented by molecules **1** (polar X−H), **6** (aromatic C−H), **7** (alkyl C−H), **8** and **5** (any C−F), **9** (nitrile), and **3** (carbonyl). **1**, **6** and **7** were used to compare differently polarized H atoms; **8**, **5**, **9** and **3** were used to model the negative potential of lone‐pair and/or π electrons. Molecules **10**–**12** (bottom in Figure 1) are additional lone‐pair bearing molecules used for computational evaluations involving a lone‐pair on C (**10**), O (**11**) and N (**12**).^[38]^

The interactions between the central and contact groups are anticipated to be mainly electrostatic in nature,[^2^, ^3f^, ^8^, ^39^] with a possible contribution of donor‐acceptor orbital interactions[^35^, ^40^] and dispersion (particularly for aromatic molecules).^[41]^

As it is known that the distribution of electron density visualized by the Molecular Electrostatic Potential Maps (MEPs) are good indicators for the (directionality of) electrostatic interactions,[^16f^, ^42^] the MEPs of each molecules is given in Figure 1 (all plotted at the same colour scale). The LUMOs of the central groups **1**–**5** as well as the HOMOs of the other molecules are shown to anticipate possible interactions between these frontier molecular orbitals (see Figure S1 for all the HOMOs and LUMOs).

For molecules **1**–**5** it can be seen that the location of the LUMOs roughly coinside with the loction of the anticipated σ/π‐hole indicated by the MEPs. The π‐hole of nitromethane **4** **a** is poorly visible on this colour scale as it blends in with the polarized H‐atoms; the π‐hole is clearly visible when plotted at a different colour scale (see Figure S2). While the LUMOs of **2**–**4** are hardy located on the hydrogen atoms, the highest potential on the MEPs of **2**–**4** is actually found on their hydrogen atoms. This suggests that these molecules might have a dual interaction preference with lone‐pair or π electrons (the IsoStar analysis and DFT computations might elucidate the preference).

The positive potential of the anticipated σ/π‐holes (in kcal⋅mol^−1^) in the model central groups decrease in the order of **1** (+59)>**4a** (+32)>**5** (+28)>**2** (+25)>**3** (+22)>**4b**=**6** (+19). Interstingly, tetrafluoromethane **8** (used to model a lone‐pair on F) displays a similar σ‐hole potential as the π‐hole on nitromethane **4** **a** (+32 kcal⋅mol^−1^). Trifluoromethane moieties are indeed known as σ‐hole donors, although they are hardly directional, probably due to steric and electronic repulsion that the three F‐atoms impose on any interacting electron rich entity (see also Figure S3).^[43]^ The N‐ and O‐centered lone‐pairs on **1**, **3**, **9**, **11** and **12** (and also **4**, see Figure 1) are most negative at about −40 to −45 kcal mol^−1^, followed by the π‐electrons in benzene **6** (−20 kcal mol^−1^), the C‐centered lone‐pair of carbon monoxide **10** (−16 kcal⋅mol^−1^) and the lone‐pair electrons of F in **5** and **8** (>−10 kcal⋅mol^−1^). Propane (**7**) is hardly polarized (−3 to +11 kcal mol^−1^) and the extremes are not descernable on the color scale used (see Figure S2 for a MEP with alternative scale). Interestingly, the C‐atoms of propane (**7**) are most electronegative, suggesting they might act as the *de facto* ‘electron rich’ site.[^35b^, ^44^] Most of the HOMOs of **1**, **3**, **6**, **8** and **10**–**12** coinside with the most negative site of the MEPs, which in turn corresponds to a lone‐pair or π electrons. This is not the case for **5** and **9**, where the HOMO is (mostly) a C−C π‐bonding orbital, while the most electron rich sites are found on N/F. The lone‐pair electrons of the F‐atoms (p‐orbitals) in **5** are found as hydridized with the π electrons of the arene core in most of the bonding molecular orbitals (not shown). The lone‐pair on nitrogen in **9** is the HOMO‐2 (after the two degenerate π‐bonding orbitals).

### 5.2 DFT Calculations of Model Adducts

**Figure 2:**
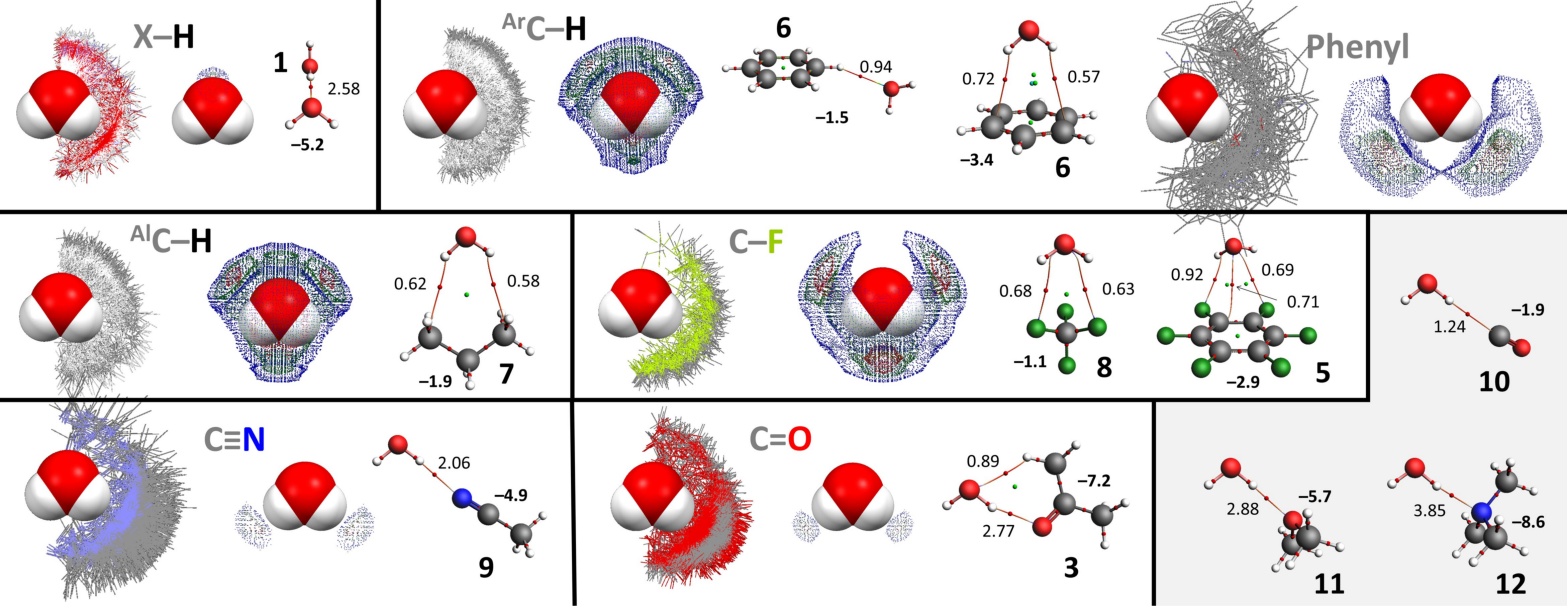
IsoStar plots of data involving water as ‘central group’ (in space filling mode). In one plot, the central group is surrounded by the specified ‘contact groups’ (in wireframe mode, only the asymmetric data is shown) where any of the atoms of both groups are within each other's van der Waals radii; in the other plot the density of the contact point of the ‘contact group’ (H, ring centroid, F, N, O) is shown increasing from blue<green<red. Also shown are the DFT geometry optimized structures of water adducts, together with their ΔE^BSSE^ (in kcal mol^−1^, see also Table 2) and the atoms‐in‐molecules analysis with bond paths (thin red lines) and bond critical points (red spheres, with density *ρ* in a.u. x 10^2^). The bottom right (grey background) only shows DFT geometries as there was not enough data in the IsoStar database for a comparison.

**Figure 3:**
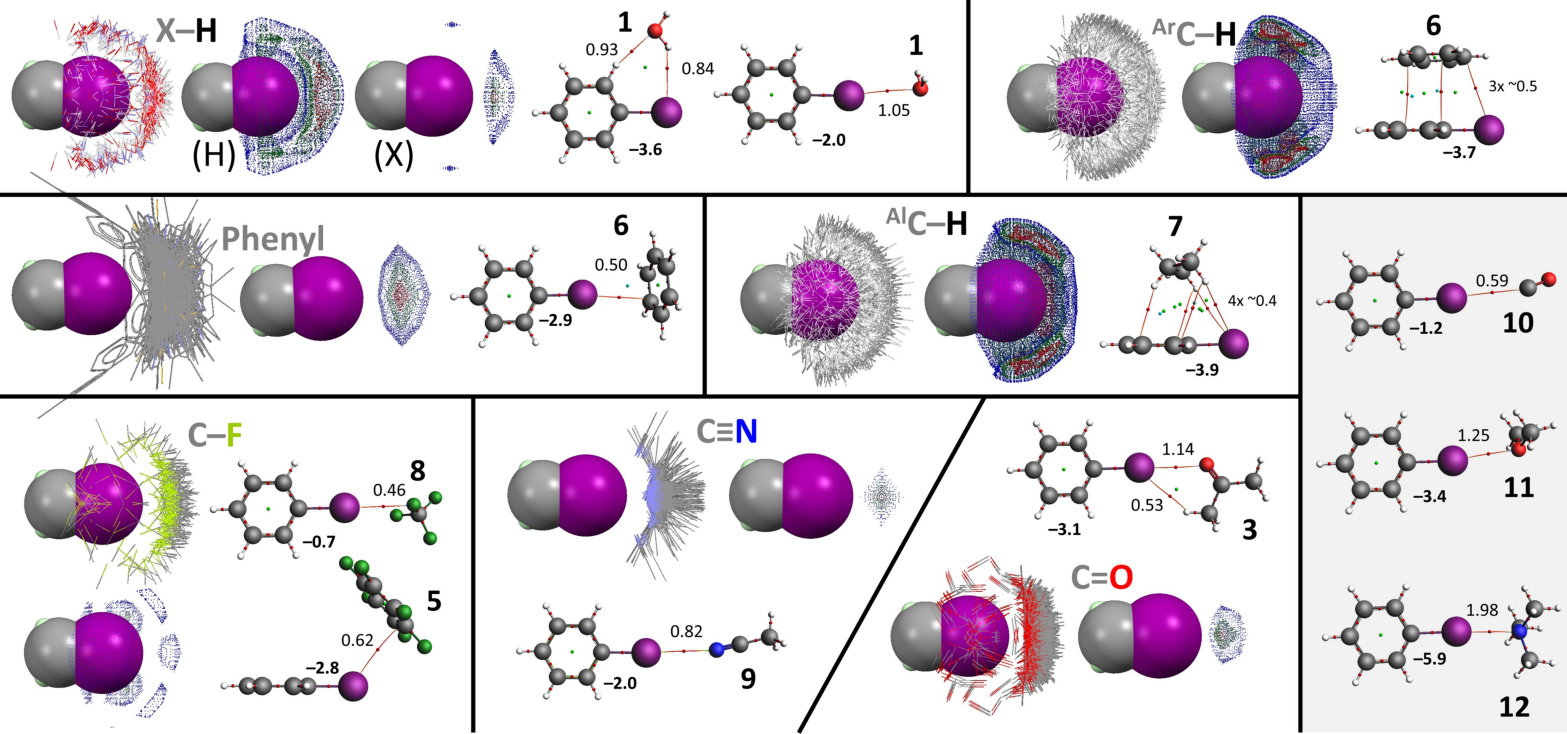
IsoStar data involving an aromatic iodide as ‘central group’ (in space filling mode) and model DFT calculations with iodobenzene (**2**). See caption of Figure 2 for general details. For any polar X−H, the IsoStar density plots shown are with H or X (X can be O, N, or S). All the IsoStar plots were symmetry expanded. See also Figure S8 for the IsoStar data with aromatic bromines.

**Figure 4:**
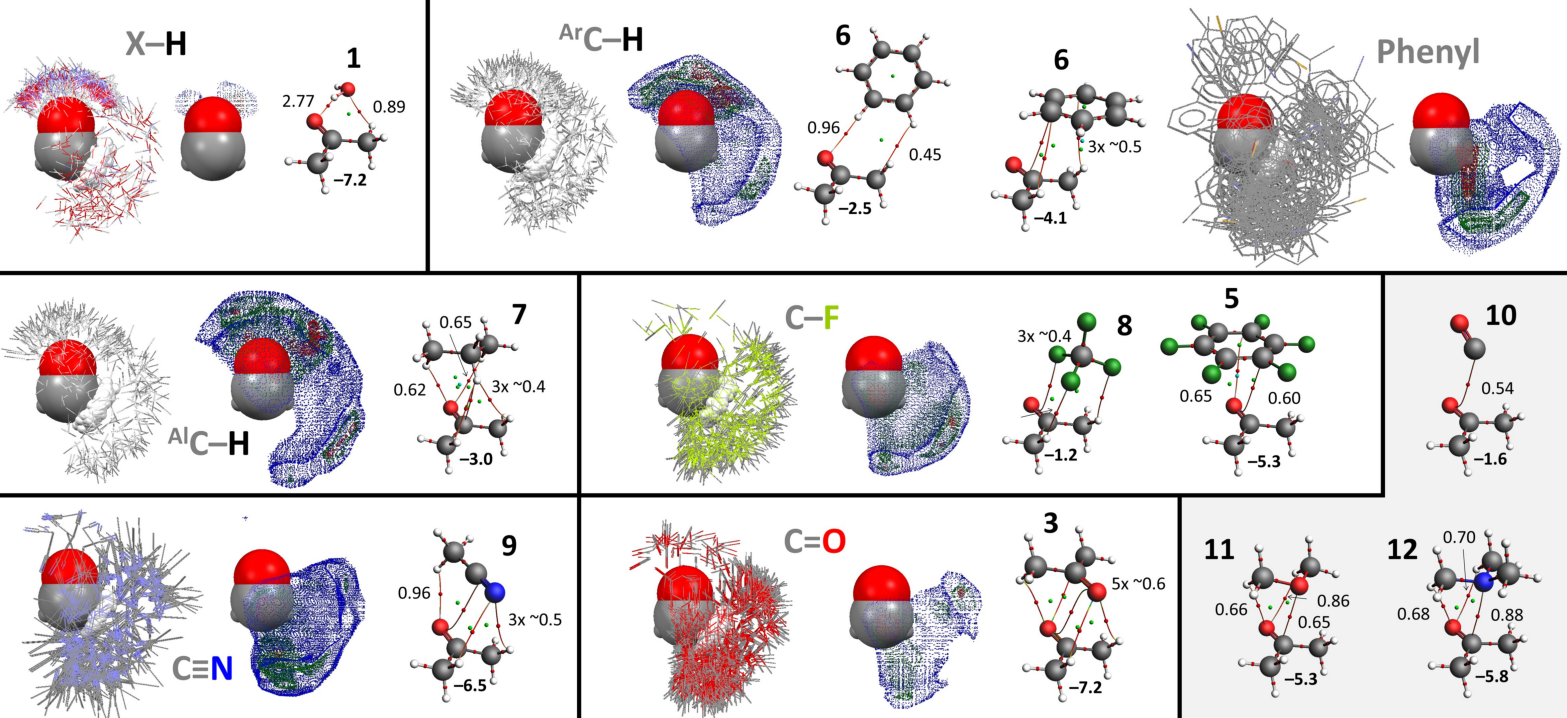
IsoStar data involving an acetyl as ‘central group’ (in space filling mode) and model DFT calculations with acetone (**3**). See caption of Figure 2 for general details.

**Figure 5:**
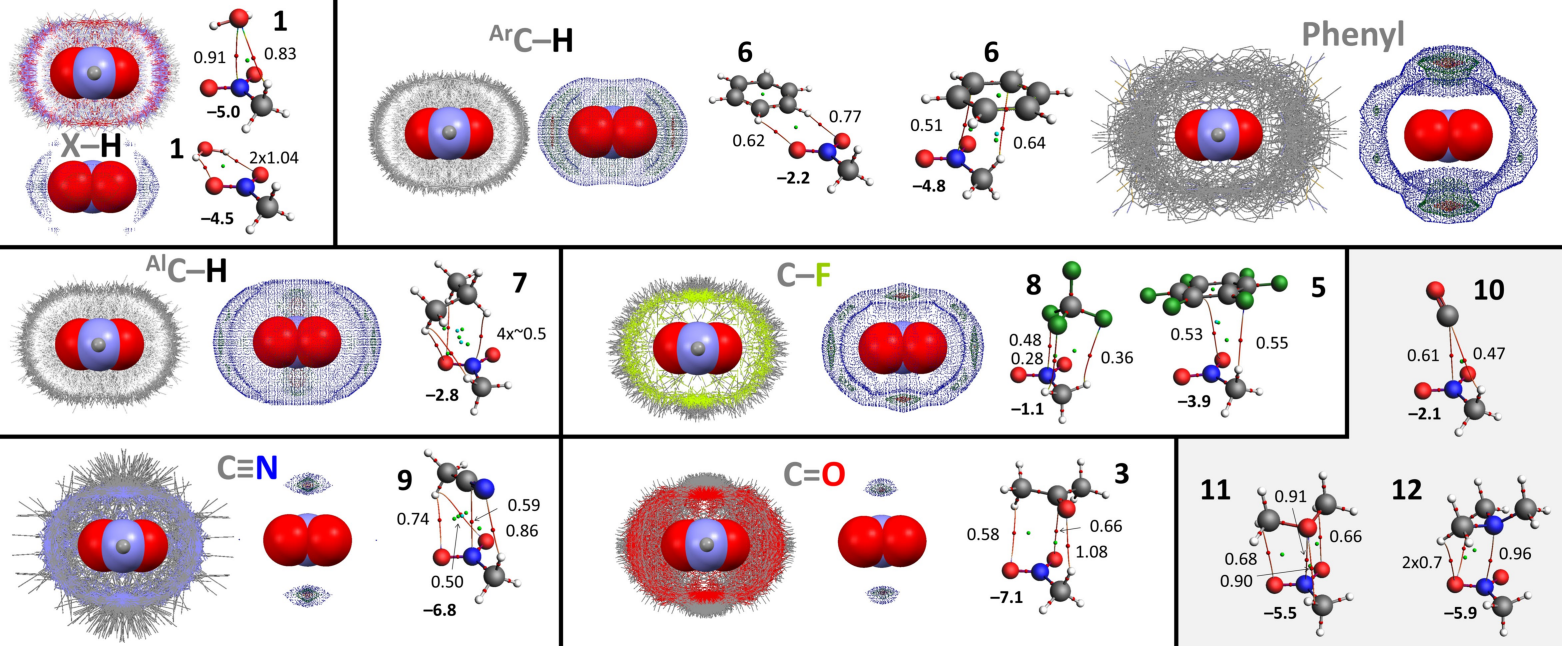
IsoStar data involving any nitro moiety as ‘central group’ (in space filling mode) and model DFT calculations with nitromethane (**4** **a**). The computations involving nitrobenzene (**4** **b**) can be seen in Figure S9. See caption of Figure 2 for general details. All the IsoStar plots were symmetry expanded.

**Figure 6:**
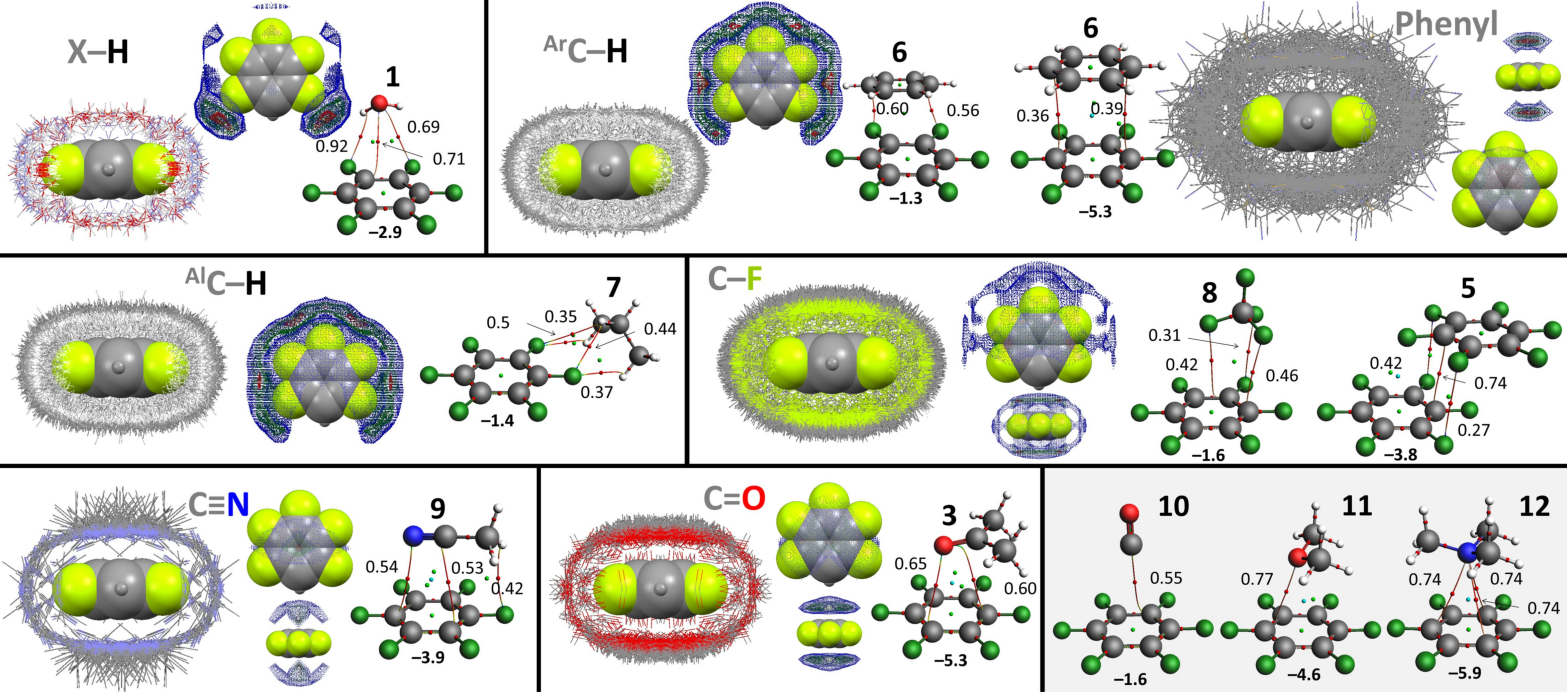
IsoStar data involving a pentafluorophenyl ring as ‘central group’ (in space filling mode) and model DFT calculations with hexafluorobenzene (**5**). See caption of Figure 2 for general details. All the IsoStar plots were symmetry expanded.

**Figure 7:**
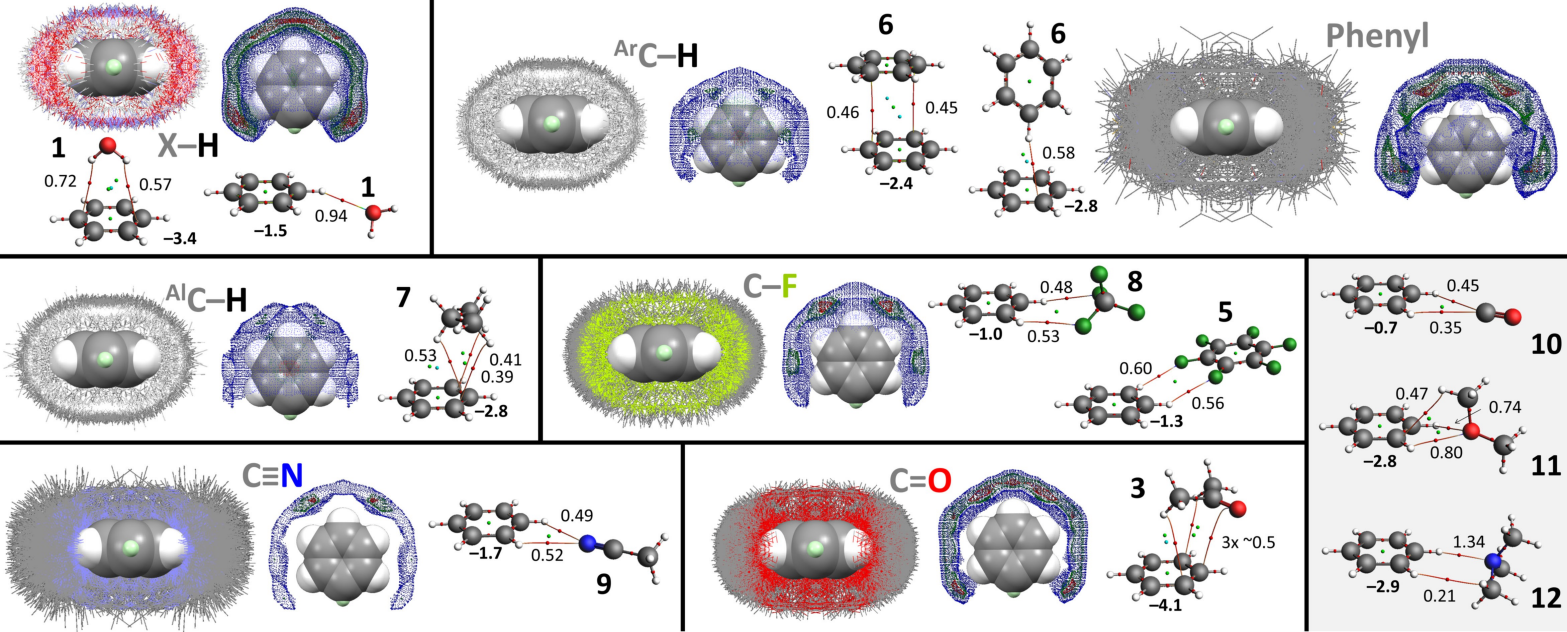
IsoStar data involving a phenyl ring as ‘central group’ (in space filling mode) and model DFT calculations with benzene (**6**). See caption of Figure 2 for general details. All the IsoStar plots were symmetry expanded.

The numerical results of adducts calculated between the models **1**–**6** of the IsoStar ‘central groups’ and models **1**, **3**, **5**–**9** of the ‘contact groups’ are given in Table 2. Also shown are the data for adducts between **1**–**6** and molecules **10**–**12** used as models for a lone‐pair on C (**10**), O (**11**), and N (**12**). The energies given are corrected for the basis set superposition error (ΔE^BSSE^) using the counterpoise method and an energy decomposition analysis is also given as percentages of electrostatic attraction/orbital interactions/dispersion (based on ΔE, not ΔE^BSSE^). Energy decomposition analyses of small molecular adducts[^39^, ^40d^, ^40e^, ^40f^] have proven to be useful to probe the physical origins of hydrogen[^35^, ^40a^] and halogen[^40b^, ^40c^] bonding. The interaction energies as well as the optimised geometries are shown in Figures 2–7 alongside the IsoStar database analyses. Prior to a more detailed discussion, some general observations from the entire dataset are presented here.

**Table 2:** Numerical overview of the interaction energies ΔE^BSSE^ (corrected for basis set superposition error) for molecules used to model the central group (**1**–**6**) and contact group (**1**, **6**, **8**, **5**, **9**, **3**) in the IsoStar analysis. For comparison purposes, adducts of **1**–**6** were also computed with molecules **10**–**12**. The cells are color coded based on ΔE^BSSE^ in six 1.5 kcal mol^−1^ increments from 0 (green) to −9 kcal mol^−1^ (red). Also given for each adduct is the energy decomposition analysis of ΔE (not BSSE corrected) into electrostatic attraction/orbital interactions/dispersion. A gaphical rendering of each entry is also shown in Figures 2–7 for the adducts with **1**–**6** respectively. The type of interaction of each entry is indicated in superscript.

	σ‐hole Donors		π‐hole Donors				Reference
	**1** (H_2_O)	**2** (C_6_H_5_I)	**3** (Me_2_C=O)	**4 a** (MeNO_2_)	**4 b** (PhNO_2_)	**5** (C_6_F_6_)	**6** (C_6_H_6_)
**1** (H_2_O)	−5.2^HB^ (65/29/6)	−3.6^HB^ (50/28/23)	−7.2^HB^ see [**1**⋅⋅⋅**3**]	−4.5^HB^ (66/17/17)	−4.9^HB^ (66/18/17)	−2.9^HB^ see [**1**⋅⋅⋅**5**]	−1.5^HB^ see [**1**⋅⋅⋅**6**]
		−2.0^Hlg, a^ (59/23/18)		−5.0^πB/HB^ (66/13/21)		−2.8^πB^ 54/14/32	−3.4^H−π^ see [**1**⋅⋅⋅**6**]
**6** (C_6_H_6_)	−1.5^HB^ (55/20/25)	−2.9^Hlg−π^ (28/16/55)	−2.5^HB^ (38/22/41)	−2.2^HB^ (47/16/36)	−2.3^HB^ (46/18/37)	−1.3^HB^ (29/15/56)	−2.8^HB^ (32/15/53)
	−3.4^H−π^ (41/22/37)	−3.7^πB^ (30/12/58)	−4.1^H−π/πB^ (35/16/49)	−4.8^πB^ (41/16/43)	−4.7^πB^ (33/11/56)	−5.3^πB^ (36/11/53)	−2.4^πB^ (30/12/58)
**7** (propane)	−1.9^OG^ (34/25/41)	−3.9^H−π^ (28/13/59)	−3.0^HB^ (28/17/55)	−2.8^HB^ (32/20/48)	−3.7^HB/πB^ (27/13/60)	−1.4^HB^ (22/13/65)	−2.8^H−π^ (27/14/59)
**8** (CF_4_)	−1.1^HB^ (48/15/37)	−0.7^Hlg^ (38/14/48)	−1.2^HB/πB^ (18/17/65)	−1.1^πB/HB^ (16/19/66)	−1.8^πB/HB^ (32/12/55)	−1.6^πB^ (20/18/62)	−1.0^HB^ (28/15/56)
**5** (C_6_F_6_)	−2.9^HB^ (56/15/30)	−2.8^Hlg^ (43/13/44)	−5.3^πB^ (34/18/48)	−3.9^πB/HB^ (32/18/50)	−5.3^πB^ (27/15/59)	−3.8^πB^ (21/20/60)	−1.3^HB^ see [**5**⋅⋅⋅**6**]
**9** (MeC≡N)	−4.9^HB^ (65/29/6)	−2.0^Hlg^ (52/27/20)	−6.5^πB^ (56/17/27)	−6.8^πB/HB^ (57/17/27)	−5.5^πB/HB^ (49/15/36)	−3.9^πB/HB^ (36/18/46)	−1.7^HB^ (39/17/44)
**3** (Me_2_C=O)	−7.2^HB^ (62/29/9)	−3.1^Hlg^ (52/25/23)	−7.2^πB^ (50/17/33)	−7.1^πB/HB^ (53/17/30)	−5.6^πB/HB^ (43/16/42)	−5.3^πB^ see [**3**⋅⋅⋅**5**]	−4.1^H−π/πB^ see [**3**⋅⋅⋅**6**]
							
**10** (C≡O)	−1.9^HB^ (56/33/11)	−1.2^Hlg^ (42/27/32)	−1.6^πB^ (36/18/46)	−2.1^πB/HB^ (48/19/33)	−1.4^πB^ (40/17/44)	−1.6^πB^ (37/22/42)	−0.7^HB^ (29/25/46)
**11** (OMe_2_)	−5.7^HB^ (61/29/10)	−3.4^Hlg^ (52/21/27)	−5.3^πB^ (44/17/39)	−5.5^πB/HB^ (51/16/33)	−4.7^πB/HB^ (44/16/40)	−4.6^πB^ (38/16/47)	−2.8^HB^ (45/15/40)
**12** (NMe_3_)	−8.6^HB^ (59/32/9)	−5.9^Hlg^ (56/26/18)	−5.8^πB^ (43/18/38)	−5.9^πB/HB^ (50/17/33)	−6.1^πB/HB^ (43/17/39)	−5.9^πB^ (41/14/46)	−2.9^HB^ (45/21/33)

**HB**=hydrogen bonding geometry with lone‐pair electrons; **H‐π**=hydrogen bonding geometry with π electrons; **Hlg**=halogen bonding geometry with lone‐pair electrons; **Hlg‐π**=halogen bonding geometry with π electrons; **πB**=π‐hole bonding (or stacking) geometry; **OG**=other geometry.^**a**^ similar geometry with pentafluoroiodobenzene (not shown) gave an adduct with ΔE^BSSE^=−3.5 kcal mol^−1^ and energy decomposition analysis of ‘65/27/8’.

The interaction energies (ΔE^BSSE^) are all negative and vary considerably: from –0.7 kcal mol^−1^ in [**2**⋅⋅⋅**8**] and [**6**⋅⋅⋅**10**] to –8.6 kcal⋅mol^−1^ in [**1**⋅⋅⋅**12**]. Of the two σ‐hole donors water (**1**) and iodobenzene (**2**), water generally forms the most stable adducts. Iodobenzene adducts are least stable, also compared to the π‐hole donors (**3**–**5**). The most potent of the π‐hole donors considered are acetone (**3**) and nitromethane (**4** **a**). The interaction energies involving the reference molecule benzene (**6**) are typically the smallest.

For adducts with water **1** (first row) and benzene **6** (second row) as model ‘contact groups’ two distinct geometries were considered; one where an O−H or C−H hydrogen is involved in a hydrogen bonding interaction with a lone pair of electrons in **1**–**5** (superscripted ‘HB’) and some other geometry (halogen‐, π‐hole or H‐π bonding superscripted as ‘Hlg’, ‘πB’ or ‘H‐π’). For adducts with water, the hydrogen bonding geometry is most stable with **1**, **2**, **3**, **4** **b** and **5**. For the water dimer there is no other possibility than a hydrogen bond. Calculations of the water adducts with acetone (**3**) and nitrobenzene (**4** **b**) were also started from a π‐hole bonding geometry, but both converged to the hydrogen bonding geometry. Interestingly, the π‐hole bonding geometry in [**4** **a**⋅⋅⋅**1**] is 0.5 kcal mol^−1^ more stable than the hydrogen bonding geometry. This is likely due to the π‐hole interaction aided by an additional stabilisation by a C−H⋅⋅⋅OH_2_ hydrogen bonding interaction (see also Figure 5). Also interesting to note is that the hydrogen and π‐hole bonding geometries involving hexafluorobenzene (**5**) are nearly identical in strength, while the OH_2_⋅⋅⋅π interaction with benzene is more than twice as favourable as the C−H⋅⋅⋅OH_2_ interaction. In the case of benzene (**6**) adducts represented in the second row, the hydrogen or halogen bonding geometry is weaker than the π‐bonding geometry (except for the benzene dimers). The most stable (−5.3 kcal mol^−1^) is the π–π stacking interaction with hexafluorobenzene ([**5**⋅⋅⋅**6**]) in which the distribution of electron density is opposite. This large interaction energy is unsurprising given that the crystal structure of the [**5**⋅⋅⋅**6**] adduct is known to consist of alternating π‐π stacked pillars,^[45]^ and that mixing both liquids (m.p. **5**=5.0 °C;^[46]^ **6**=5.5 °C)^[47]^ gives a solid at <20 °C under the release of heat (m.p. [**5**⋅⋅⋅**6**]=25 °C).^[48]^

Adducts where model contact groups **7**, **8**, and **10** act as Lewis base, as well as the hydrogen bonding geometries with **6**, are generally the weakest and nearly always dominated by dispersion. Interactions with the other model ‘contact groups’ **1**, **3**, **9**, **11** and **12** are typically more stable and dominated by electrostatics. This is in line with the relatively large negative potential of **1**, **3**, **9**, **11** and **12** (−41 to −45 kcal mol^−1^) compared to **6**, **7**, **8** and **10** (−3 to −20 kcal⋅mol^−1^). The interaction energy of adducts of **1**–**6** with **10** (CO), **11** (OMe_2_) and **12** (NMe_3_) increases in the order of electronegativity on the lone‐pair of electrons on C, O and N respectively (see Figure 1). All these observations confirm earlier reports that molecular adducts become dominated by electrostatics (over dispersion) when the molecules are more polarized and that more polar molecules form more stable adducts.[^13^, ^41c^, ^49^]

Orbital interactions are never the dominant force, and fluctuate between 11 % in [**4** **b**⋅⋅⋅**6**] to 33 % in [**1**⋅⋅⋅**10**]. This suggests that all the adducts are closed‐shell interactions, which raises the question whether orbital interactions of the donor‐acceptor kind are relevant at all in the adducts considered. Thus, a closer scrutiny of the orbital interactions involving NMe_3_ (**12**) is included in the supporting information and summarized in Table S1. Visual inspection of the molecular orbitals of the adducts (see Figures S4–S6) make it clear that some of these originate from *both* molecular fragments. The contributions are modest however, as one of the original orbitals typically contributes less than about 2 %. These small orbital interactions with **12** are of the donor‐acceptor kind involving **1** and **2** (Figure S4), **3** and **4** (Figure S5), but not with **5** (Figure S6a). However, such interactions are of the donor‐acceptor kind when using a stronger base such as cyanide anion (^–^C≡N, Figure S6b) and when using a more electron deficient ring such as trifluoro‐s‐triazine (Figure S6c).^[50]^ It must be noted that orbital interactions involving the HOMO and LUMO orbitals can become a dominant driving force in adducts involving better σ‐ or π‐hole donors/acceptors. This is exemplified by the anionic adducts [Cl^–^⋅⋅⋅I−Ph (**2**)]^−^ (−15.8 kcal mol^−1^) and [N≡C^−^⋅⋅⋅*p*‐benzoquinone (**13**)]^−^ (−24.2 kcal mol^−1^); both are driven for about 50 % by orbital interactions (Table S1) and there is a clear orbital interaction between the HOMO of the anion and the LUMO of the σ‐ or π‐hole donor (Figure S7). Such interactions are relatively rare in the CSD, making a reliable IsoStar analysis difficult.^[51]^ However, the HOMOs and LUMOs shown in Figure 1 are still indicative of the directionality of an interaction with these σ‐ and π‐hole donors and acceptors, provided one or both interacting partners are more polarized (e. g. charged).

### 5.3 IsoStar Analysis Compared with DFT Calculations and AIM Analyses

The IsoStar plots involving the various ‘central groups’ **1**–**6** are shown in Figure 2–7. For each ‘central group’, a plot is provided involving van der Waals overlap with any atom of the ‘contact group’, as well as a density plot (where increased density is colour coded blue<green<red). Also provided are the ball‐and‐sticks molecular models of geometry optimized adducts between a model central group and the model contact groups **1**, **3**, **5**–**9** as well as **10**–**12** (used as a comparative series of lone‐pairs, see also Table 2). The interaction energies (ΔE^BSSE^ in kcal mol^−1^, bold font) and densities (*ρ* in a.u. x 10^2^) of the bond critical points (bcp) of an atoms‐in‐molecules analysis are also shown alongside the adducts.

The data involving water as central group is show in Figure 2. In the top‐left the IsoStar data is shown where any polar hydrogen (X−H, where X=S, O, N) is within the sum of the van der Waals radii of H (the contact group) and O (the central water group). The contour density plot of the H‐atoms of the contact groups is depicted adjacent the IsoStar data and is clearly concentrated at the locations of the lone‐pair electrons of water. The water dimer shown in ball‐and‐stick representation is the geometry obtained after DFT optimization. The ΔE^BSSE^ of this dimer is −5.2 kcal mol^−1^ and the atoms‐in‐molecules analysis reveals a single bond critical point (small red dot) with *ρ*=2.58×10^−2^ a.u.. The data with other contact groups are similarly configured.

It is noteworthy that the most stable adducts (ΔE^BSSE^≥5 kcal mol^−1^) involve the most polar **1**, **3** and **9**, which is also reflected in the tight grouping of data observed in the IsoStar plots with **1**, **3** and **9**. A similar grouping, but less tight, is observed for the O−H⋅⋅⋅π bonding geometry of the [water⋅⋅⋅benzene (**6**)] adduct. The locations of these groupings coincide with the typical idea of a hydrogen bonding interaction.[^3b^, ^4b^, ^52^]

The interactions with aromatic (**6**) and alkyl (**7**) C−H are much weaker at <2 kcal mol^−1^ and the IsoStar plots suggest far less directionality than with **1**, **3** and **9**. Contrary to the IsoStar plot with polar X−H (X=N, O, S), the density of contact groups with **6** and **7** are not located near the lone‐pair electrons of water. Instead, there are two patches of density near O at the extension of the H–O vectors, and one rather large patch in between both H‐atoms *perpendicular to* the H_2_O plane. These locations were not reproduced by DFT: with benzene (**6**) a C−H⋅⋅⋅OH_2_ geometry could be optimized and with propane (**7**), the energy minimum geometry involves a OH_2_⋅⋅⋅C−H interaction (also when starting from various C−H⋅⋅⋅OH_2_ geometries). In the case of any C−F contact group, a nearly identical grouping as with C−H is observed, although here some density is also present in between both H's and *in* the H_2_O plane. The model calculations with CF_4_ (**8**) and C_6_F_6_ (**5**) both converged at a geometry that is more like weak O−H⋅⋅⋅F hydrogen bonding than the densities observed in the IsoStar plots. Notably, with C−F as well as with both C−H contact groups there is a lack of density near H at the extension of the O−H bond. For both C−H's this observation can be understood in terms of H⋅⋅⋅H repulsion; for the C−F contact groups this could be rationalized based on a (small) σ‐hole on F (although some density around the H's was anticipated).

The bcp's of the computed adducts are generally densest for the most stable adducts, especially those dominated by electrostatics (see Table 2). This is most obvious in the comparative series with only one bcp: the C−H⋅⋅⋅OH_2_ geometry with benzene (**6**) < carbon monoxide (**10**) < acetonitrile (**9**) < water (**1**) < dimethylether (**11**) < trimethylamine (**12**). Similar observations can be made for the adducts with the other central groups (see Figures 3–7), which is consistent with other reports that mention a relationship between the density of a bcp and the interaction energy of an adduct.[^20^, ^42g^, ^53^]

For the data involving an aromatic iodide as central group (Figure 3) it is noteworthy that the tightest grouping involves the halogen bonding geometries with X of polar X–H, the π‐electrons of phenyl rings and the lone‐pair electrons in nitriles and carbonyls. This is in line with the general idea of a halogen bonding interaction,[^3c^, ^3e^, ^4b^, ^54^] as further evidenced by the relatively dense (>0.5) bcp's observed along the C−I vectors in adducts of iodobenzene with **1**, **3**, **6** and **9**.

The hydrogen bonding C−I⋅⋅⋅H−X interactions are scattered around the entire I‐atom, although some grouping of H is noticeable at the extension of the C–I vector. This is likely an artefact caused by the (apparently) much more directional C−I⋅⋅⋅X−H halogen bonding interacting. That the hydrogen bonding geometry calculated for [**1**⋅⋅⋅**2**] is 1.6 kcal⋅mol^−1^ more stable than the halogen bonding geometry, can be ascribed to the presence of two hydrogen bonding interactions (*versus* only one halogen bonding interaction). These C−I⋅⋅⋅HO and C−H⋅⋅⋅OH_2_ hydrogen bonding interactions might each contribute about 1.8 kcal⋅mol^−1^, which is slightly less than the halogen bonding geometry. That the observed distribution of X−H fragments is consistent with the halogen bonded geometry rather than the hydrogen bonded geometry is likely because of steric hindrance of the X‐group (the OH group in water will be relatively rare).

The hydrogen bonding interactions with aromatic and aliphatic C−H moieties display a grouping predominantly around the I‐atoms with a noticeable lack of data along the C–I vector. These groupings trace the distribution one might anticipate base on the MEP of **2** (Figure 1). The most stable geometries optimized with benzene (**6**) and propane (**7**) converged to structures with a similar energy of ΔE^BSSE^≈−3.8 kcal mol^−1^. This can be ascribed to the large contact surface for the π–π stacking geometry of [**2**⋅⋅⋅**6**] and the multiple CH‐π interactions in [**2**⋅⋅⋅**7**], which is also reflected in the large (∼60 %) contribution of dispersion to the total energy (see Table 2).^[41f]^ The IsoStar data for any C−F contact group is skewed to an overall geometry with a C−I⋅⋅⋅F angle around 180°, although the data is not very concentrated according to the density plot. This is consistent with the optimized structure with CF_4_ (**8**), which is hardly favourable (−0.7 kcal mol^−1^). The adduct with hexafluorobenzene [**2**⋅⋅⋅**5**] is much more stable at −2.8 kcal mol^−1^, but this larger energy likely stems from a lone‐pair⋅⋅⋅π interaction with the electron deficient **5**.[^14b^, ^14d^, ^27a^] Nearly identical groupings of data was found around the more numerous but less polarized aromatic bromines (see Figure S8)

As with water adducts, the halogen bonding geometries between **2** and **10**–**12** are more stable for more electron right lone‐pairs (as also reflected in the denser bcp's).

The IsoStar plots involving a terminal acetyl group and the DFT optimized adducts involving acetone (**3**) are shown in Figure 4. The density plots involving X–H and (aromatic or aliphatic) C−H all indicate a preference for a C=O⋅⋅⋅H hydrogen bonding interaction, with the tightest grouping observed for X−H. For all the other IsoStar plots, the majority of data is found above the carbonyl C and slightly skewed towards the methyl group. These groupings are in line with the Bürgi‐Dunitz trajectory[^15a^, ^55^] and are consistent with a π–hole bonding geometry, possible aided by additional C−H⋅⋅⋅π/N/O/F interactions.

As with **1** and **2**, the adducts between **3** and **10**–**12** are more stable for more electron right lone‐pairs (also reflected in the denser N⋅⋅⋅C/O/N bcp's). The interaction energies with **10**–**12** are in line with those computed with **1** and **2**, but the dispersion component of the adducts with **3** is relatively large (∼40 % *versus* ∼10 % with **1** and ∼25 % with **2**).

The data involving any nitro moiety along with the geometry optimized adducts with nitromethane (**4** **a**) are shown in Figure 5 (see Figure S9 for the geometry optimized adducts with nitrobenzene **4** **b**).

A preference for N=O⋅⋅⋅H hydrogen bonding interactions are observed in the density plots involving X–H and (aromatic or aliphatic) C−H, with the tightest grouping observed for the most polar X–H. Interestingly, the π‐hole bonding geometry with a water molecule is 0.5 kcal⋅mol^−1^ more stable than the H‐bonding geometry involving two N=O⋅⋅⋅H–O hydrogen bonds. Some stability of the π‐hole bonding geometry can be understood by an additional hydrogen bonding interaction with the (polar) α‐hydrogen of the methyl group. Such a C−H⋅⋅⋅OH_2_ hydrogen bonding interaction is undoubtedly weaker than a N=O⋅⋅⋅HO hydrogen bond. In fact, a single N=O⋅⋅⋅HO hydrogen bond can be estimated at −2.25 kcal mol^−1^ (−4.5/2), while the C−H⋅⋅⋅OH_2_ interaction must be less favourable than −2.5 kcal⋅mol^−1^ (−5/2). This implies that the O_2_N⋅⋅⋅OH_2_ π‐hole bonding interaction is stronger than a single N=O⋅⋅⋅HO hydrogen bond. That the π‐hole bonding geometry is not preferred in the IsoStar plot with **1** is likely because most nitro fragment are aromatic (∼28.000 CIFs) and that most aliphatic structures (∼5.000 CIFs) have at least one α‐H replaced by some other (larger) group that might sterically hinder the π‐hole bonding geometry.

For the other IsoStar plots involving a phenyl, C−F, C≡N or C=O contact group the majority of data is found above the nitro N‐atom. This is in line with the general idea of π‐hole bonding with nitro‐groups.[^16a^, ^16b^, ^16c^, ^16d^, ^16e^, ^16f^, ^16h^, ^16i^, ^56^] The groupings with nitriles and carbonyls are very tight and on par with those found for the hydrogen bonding geometries with water (Figure 2) and the halogen bonding geometries found with iodoaryls (Figure 3). The interaction energies calculated between nitromethane (**4** **a**), acetonitrile (**9**) and acetone (**3**) of about –7 kcal⋅mol^−1^ are among the largest computed with model contact groups (see Table 2). In these calculations, part of the stabilization energy can be ascribed to a hydrogen bonding interaction with the α‐H of nitromethane, which will be absent in most structures present in the CSD. Indeed, the acetonitrile and acetone adducts with nitrobenzene **4** **b** (see Figure S9) are also relatively stable (about –5.5 kcal⋅mol^−1^) without having this N/O⋅⋅⋅α‐H interaction. Similarly, the π‐hole bonding geometries between **4** and dimethyl ether (**11**) and trimethylamine (**12**) are about −5–6 kcal mol^−1^ without such N/O⋅⋅⋅α‐H interactions.

As with **1–3**, the adducts between **4** and **10**–**12** have a larger ΔE^BSSE^ and a denser N⋅⋅⋅C/O/N bcp in the order **10**<**11**<**12**. All computed energies and energy decomposition analyses with **4** are very similar to the π‐hole interactions computed with acetone (**3**, see Table 2 and Figure 4).

Shown in Figure 6 are the IsoStar plots with pentafluorophenyl rings as central group, together with the energy minimized geometries involving hexafluorobenzene. The preference for X–H and (aromatic or aliphatic) C−H contact groups clearly is a C−F⋅⋅⋅H hydrogen bonding geometry. The tightest grouping of all H‐bonding geometries is (again) observed for the most polar X−H's. This is in line with the larger interaction energy of –2.9 kcal⋅mol^−1^ obtained for [**5**⋅⋅⋅**1**] *versus* about −1.3 kcal mol^−1^ for [**5**⋅⋅⋅**6**] and [**5**⋅⋅⋅**7**]. The C−F contact groups appear broadly scattered around the pentafluorophenyl ring. Interestingly, the patches of highest density are located above/below the ring, but in particular in between two of the pentafluorophenyl's F‐atoms –not so much the ring centroid. This seems at odds with earlier reports about the directionality towards the ring centre of lone‐pair‐π interactions involving pentafluorophenyl rings,[^14b^, ^14c^, ^17a^, ^27a^, ^57^] and more in line with σ‐like complexes (a preliminary stage of aromatic nucleophilic substitutions).[^50^, ^57a^, ^57d^, ^58^] In a recent report such higher‐density patches have been described as the random distribution around a pentafluorophenyl ring, resulting from mere space‐filling.^[59]^ An alternative rationalization of the observed densities for pentafluorophenyl⋅⋅⋅F−C contacts lies with the optimized structures of [**5**⋅⋅⋅**5**] and [**5**⋅⋅⋅**8**] in which for every one C−F that is located near the ring centroid, two are located close to the rings F's (see Figure S10 for several perspective views). This is particularly relevant for the [**5**⋅⋅⋅**8**] adduct. Here, three F‐atoms of CF_4_ can concurrently match three patches of electron depletion in hexafluorobenzene: one with the rings centroid and two with the electron depletions in between two ring fluorides (see the MEP in Figure 2 and also Figure S10). Either way, the directionality of the pentafluorophenyl⋅⋅⋅F−C contacts are minimal, which is in sharp contrast to the distributions observed for phenyl rings, nitriles and carbonyls as contact groups. Indeed, in these cases nearly all the density is located above/below the ring and near the centroid. This is in line with earlier reports on the interaction between hexafluorobenzene and water[^57c^, ^57d^, ^57e^] and the directional character of pentafluorophenyl rings for several solvents and anions.[^14c^, ^27a^] The computed interaction energies between **5** and **3**, **6** and **9** of –4‐5 kcal mol^−1^ are relatively large, compared to −2.9 kcal⋅mol^−1^ for the water adduct with **5**. Thus, contrary to a recent assertion, π interactions involving a pentafluorophenyl ring are directional, but the directionality depends on the nature (and environment) of the electron rich entity. With lone‐pairs on charge‐neural fluorine's, hardly any directionality is observed. Phenyl π‐electrons and the lone‐pairs/π‐electrons on nitriles and carbonyls have a clear preference for the top/bottom of the ring, near the ring centroid.^[59a]^ These groupings in the IsoStar plots are not as tight as observed for the stronger hydrogen bonds (H_2_O⋅⋅⋅H−X), halogen bonds (aromatic C−I⋅⋅⋅O=C) or π‐hole interactions (RC(O)Me/RNO_2_⋅⋅⋅O=C). This can be rationalized by the relatively small energy penalty that is involved for parallel displacement of the electron rich partner, as has been reported for the anion‐π interaction in [**5**⋅⋅⋅Cl^−^]^−^.^[14c]^ Indeed, in‐plane movement of trimethylamine (**12**) away from the origin in the [**5**⋅⋅⋅**12**] adduct gives an energy penalty of merely 10 % for a 2 Å translation (which spans the entire C‐surface of the **5**, see Figure S11 for details). Moving **12** away from the origin, perpendicular to the ring plane is less tolerant, as a 0.3 Å translation causes an energy penalty of 10 %.

Similar to the computational results of adducts between **1**–**4** and **10**–**12**, the order in ΔE^BSSE^ and bcp density is clearly **10**<**11**<**12**. In all three [**5**⋅⋅⋅**10**–**12**] adducts, a lone‐pair of electrons it pointing towards the ring centroid, in line with earlier reported directionality of the pentafluorophenyl ring (see also Figure S10).[^14b^, ^14c^, ^27a^]

For comparison purposes, Figure 7 shows the IsoStar data with phenyl rings as central group and adducts with benzene (**6**) as a model of the phenyl rings (opposite electrostatic potential as the pentafluorophenyl ring and **5**). Surprisingly, the polar X−H contact groups are located mainly at the periphery of the phenyl ring, with only a very small density (in green) above/below the ring. This is even more surprising because the C−H⋅⋅⋅π interaction geometry is about 2 kcal⋅mol^−1^ more stable than the C−H⋅⋅⋅OH_2_ interaction geometry, and even more stable than the hydrogen bonding geometries involving **10**–**12**. On close inspection it can be seen that the IsoStar plot is dominated by O−H contact groups (O=red) around the periphery, while the top/bottom positions are dominated by N−H contact groups (N=blue). With (aromatic and aliphatic) C−H as contacting group, there is also a substantial amount of data concentrated at the phenyl ring's periphery. In these cases, however, there is also a clear increased density (in red) directly above the ring centroid (opposite to what was observed with the pentafluorophenyl ring, see Figure 6). These observations are consistent with an earlier report that found that alcohols prefer the peripheral C−H⋅⋅⋅OH interaction, while (protonated) amines and all types of C−H fragments prefer the N/C−H⋅⋅⋅π interaction geometry.^[60]^ The reason for the difference in directionality can be rationalised based on the H/lone‐pair ratio. This ratio is 1 : 2 for alcohols *versus* 2 : 1 for amines. The ratio is actually infinite (3 : 0) for protonated amines and methyl groups, which have no lone‐pair available for C−H⋅⋅⋅lone‐pair hydrogen bonding.

Phenyl rings as contact group cluster at the periphery of a phenyl central group (Figure 6). This is contrary to case of pentafluorophenyl rings, which is in line with the opposite distribution of electron density of benzene *vs* hexafluorobenzene. The clustering with phenyl rings is indicative of a T‐shaped stacking interaction. This is consistent with earlier reports^[61]^ and the computations of the benzene dimer; the T‐shaped geometry (−2.8 kcal mol^−1^) is 0.4 kcal mol^−1^ more stable than the parallel displaced geometry (−2.4 kcal⋅mol^−1^).

The lone‐pair bearing C−F, C≡N and C=O contact groups all cluster around the periphery of the central phenyl ring. This is opposite to the distributions observed with the pentafluorophenyl ring (Figure 6) and consistent with C−H⋅⋅⋅lone‐pair hydrogen bonding interactions.

The ΔE^BSSE^ and bcp density of the adducts between benzene **6** and **10**–**12** increases in the order **10**<**11**<**12**, as was the case for these adducts with **1**–**5**.

### 5.4 Concrete Examples of the CSD Structures Using Hirschfeld and DFT Analysis

**Figure 8:**
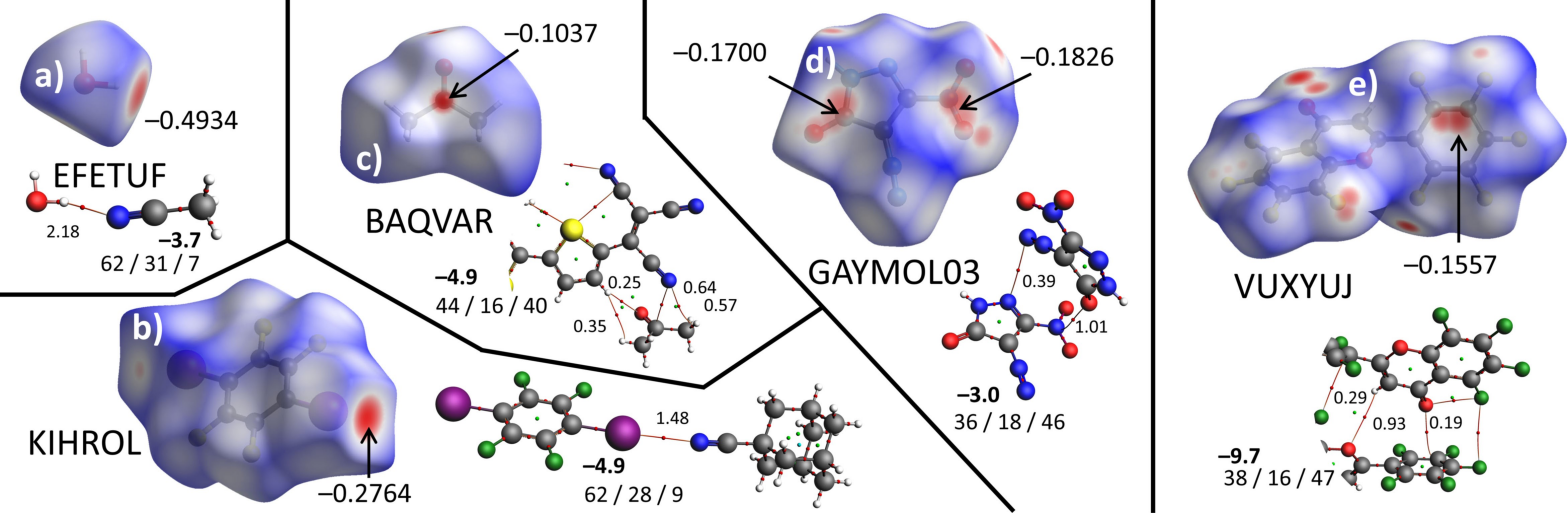
Hirshfeld surfaces with *d*_norm_ values between positive (blue) and negative (red), together with atoms‐in‐molecules and energy decomposition analysis (electrostatic/orbital/dispersive interactions) of crystal structures selected from the IsoStar datasets that contain an example of a hydrogen bond in EFETUF^[62]^ (**a**), a halogen bond in KIHROL^[63]^ (**b**), and π‐hole like interactions with a carbonyl in BAQVAR^[64]^ (**c**), a nitro group in GAYMOL03^[65]^ (**d**) and a pentafluorophenyl ring in VUXYUJ^[66]^ (**e**). The negative values in bold represent the BSSE corrected interaction energies in kcal mol^−1^. All calculations were performed using the atomic coordinates from the crystal structure at B3LYP−D3/def2‐TZ2P level of theory.

Concrete examples of crystal structures displaying an interaction involving the electron deficient central groups assessed in the IsoStar database analysis are shown in Figure 8. These examples were chosen to represent the σ‐holes water (a) and an aromatic C−I (b), and the π‐holes on a carbonyl (c), a nitro group (d) and a pentafluorophenyl ring (e). The interaction energy (ΔE^BSSE^ in bold font) and an energy decomposition analysis is provided alongside the AIM analyses computed using the atomic coordinated found in the crystal structure. Also shown for each structure is the Hirshfeld surface^[36]^ with indicated *d*_norm_ values.

All the calculated interaction energies, as well as the energy decomposition analyses and the densities of the bcp's are similar to those computed in model systems with **1**–**4** (see Table 2). The large ΔE^BSSE^ of −9.7 kcal mol^−1^ obtained for the pentafluorphenyl‐carbonyl interaction in VUXYUJ is a result of multiple polar interactions (such as a O⋅⋅⋅H−C interaction). Moreover, in all cases the location of the patches where *d*_norm_ is minimal (red) coincides with the anticipated location of the σ‐/π‐holes based on the MEPs of **1**–**6** (Figure 1). The relatively large negative *d*_norm_ values between −0.1037 in BAQVAR and −0.4934 in EFETUF signify a significant overlap of van der Waals shells,[^36c^, ^67^] which is highly indicative of bonding interaction.

## 6 Concluding Remarks

In conclusion, nearly all of the IsoStar distributions collected in Figures 2–7 follow expectations based on the electrostatic potential in the central and contact group, as visualized by the MEPs of **1**–**12** (Figure 1). The observed grouping is tightest when both the central group and the contact group are polar and complement each other's patterns. This is the case for both the σ‐hole and the π‐hole donors that were considered, which thus behave very similarly. Inspection of individual cases of interactions with a σ‐hole (hydrogen and halogen bonding) and a π‐hole (with carbonyl, nitro and pentafluorophenyl) are consistent with the IsoStar data and the computational results with models molecules **1**–**12**.

The largest degree of directionality as judged from the tightness of grouping in the IsoStar plots was typically observed for interactions with ΔE^BSSE^ of −4 to −9 kcal mol^−1^. These results thus show that for a large range of different interaction partners, a computational analysis can be a proper estimate of the directionality that such interactions can manifest in crystal structures. The value of −4 kcal⋅mol^−1^ can be taken as a benchmark for the likely utility of an interaction in crystal engineering, provided the model molecules are not too large and display mainly one type of interaction. Weaker interactions will be of less practical use to predict/engineer crystal structures, albeit they can still be important forces in, e. g. catalysis (transition state stabilization) and drug design (optimal match between drug and enzyme binding pocket). The −4 kcal mol^−1^ benchmark could be surpassed with all the σ‐ and π‐hole donors studied, provided the appropriate interacting partner.

A numerical evaluation of the clustering observed in the IsoStar plots that can be compared across central‐contact group pairs would allow a ranking of the *observed* directionality within crystal structures. Besides the −4 kcal mol^−1^ computational estimate, such a ranking could give a superior estimation of the *actual* utility of an interaction in crystal engineering. A new IsoStar‐like software program is currently being compiled with the express aim of providing such a parameter of observed directionality.

## Conflict of interest

The author declares no conflict of interest.

## Supporting information

As a service to our authors and readers, this journal provides supporting information supplied by the authors. Such materials are peer reviewed and may be re‐organized for online delivery, but are not copy‐edited or typeset. Technical support issues arising from supporting information (other than missing files) should be addressed to the authors.

Supplementary
