# Supplementary material for: DFT and IsoStar Analyses to Assess the Utility of σ‐ and π‐Hole Interactions for Crystal Engineering
Source: Chemphyschem. 2020 Dec 22;22(2):141–53. doi: 10.1002/cphc.202000927 (PMC7898519; doi:10.1002/cphc.202000927)
Supplement: Supplementary file 1 — Supplementary [file CPHC-22-141-s001.pdf]

ChemPhysChem

Supporting Information

DFT and IsoStar Analyses to Assess the Utility of σ- and π-Hole Interactions for Crystal Engineering

Tiddo Jonathan Mooibroek*

Table of contents:

Figure S1. Frontier molecular orbitals of **1**–**12**........p2
Figure S2. MEPs of **4a** and **7** on alternative energy scales........p3
Figure S3. DFT analyses (NCI, AIM, EDA) of tetrafluoromethane (**8**) adducts with **10**–**12**........p4
Figure S4. Orbital interaction in trimethylamine (**12**) adducts with **1** and **2**........p6
Figure S5. Orbital interaction in trimethylamine (**12**) adducts with **3**, **4a** and **4b**........p7
Figure S6. Orbital interaction with π-acidic rings **5** and **13**........p8
Figure S7. Orbital interaction between a simple *σ*- and *π*-hole donor and an anion........p9
Figure S8. IsoStar data of aromatic bromines........p10
Figure S9. DFT calculations with model nitrobenzene (**4b**)........p11
Figure S10. Perspective views of hexafluorobenzene (**5**) adducts with **5**, **8** and **10**–**12**........p12
Figure S11. Translating trimethylamine (**12**) in its adduct with hexafluorobenzene (**5**)........p13

Table S1. Overview and discussion of orbital interactions shown in Figures S4–S6........p5
Table S2. Cartesian coordinates of geometry optimized adducts........p14

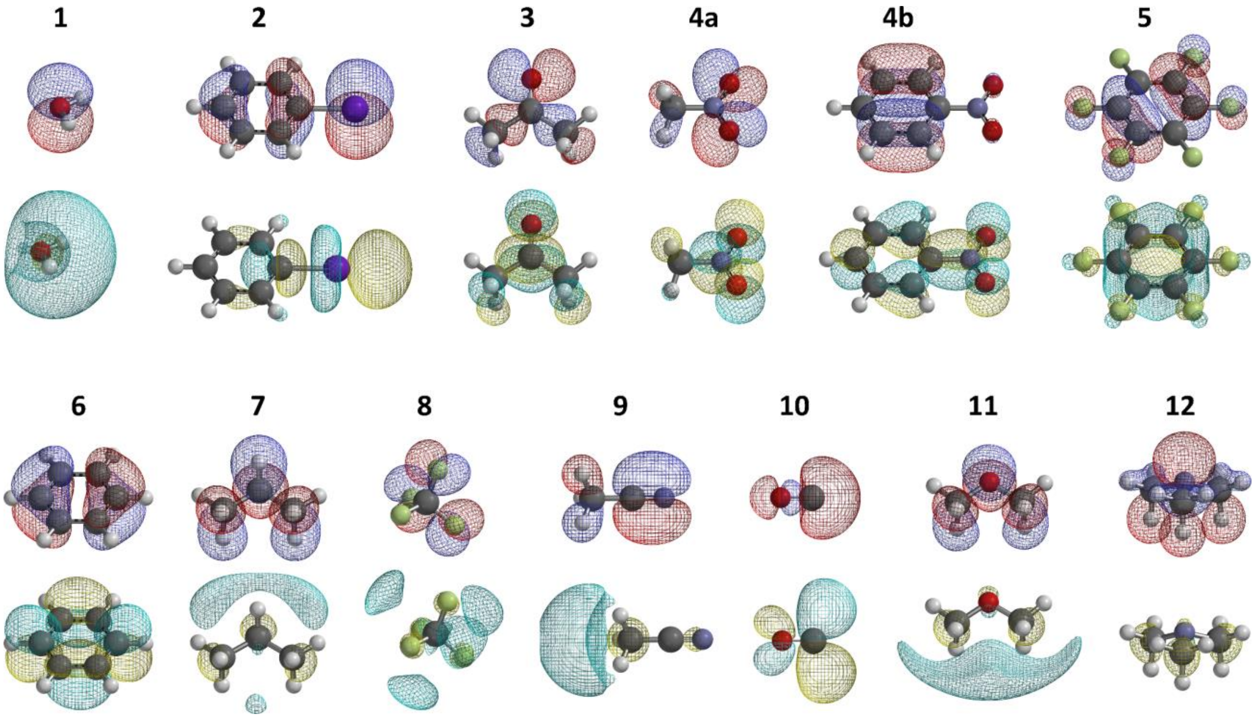

Figure S1. The HOMOs (top) and LUMOs (bottom) of molecules **1**–**12** calculated with DFT at the ωB96X-D / 6-31+G** level of theory. See also Figure 1.

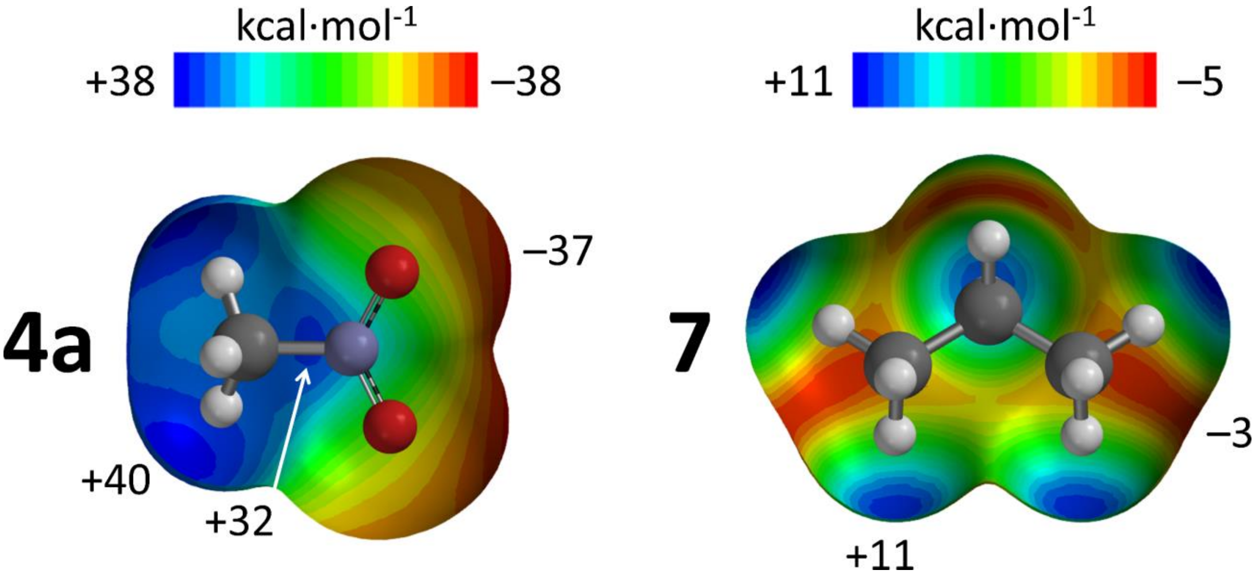

Figure S2. MEP´s of nitromethane **4a** and propane **7** computed using DFT at the ωB96X-D / 6-31+G** level of theory and plotted on the colour scale as defined above. See also Figure 1 for these MEPs on another colour scale where the polarization is less clear.

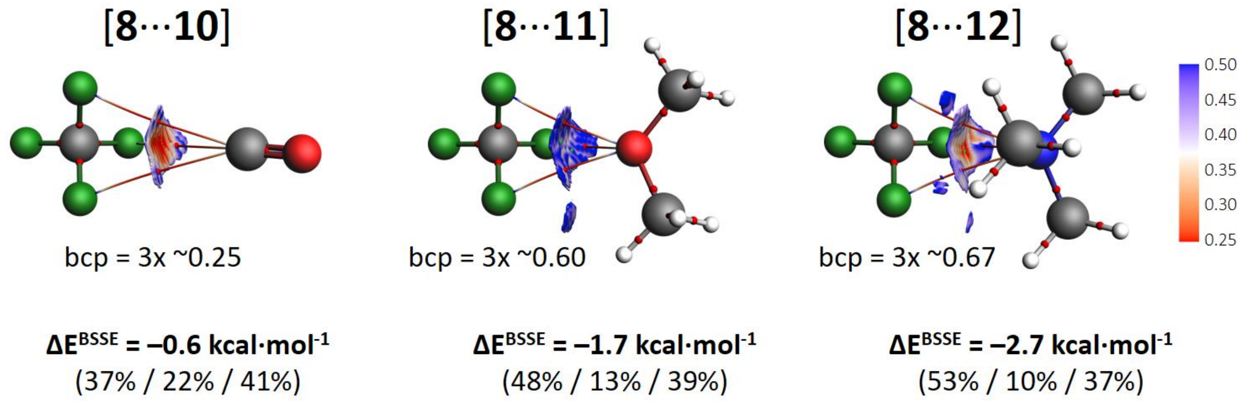

Figure S3. Representation of DFT calculations performed on adducts of tetrafluoromethane **8** acting as σ-hole for carbon monoxide (**10**), dimethylether (**11**) or trimethylamine (**12**). The geometries were optimized with Spartan at the B3LYP-D3/def2/TZVP level of theory. ADF (B3LYP-D3/TZ2P) was used to calculate: the interaction energies (ΔE^{BSSE}); the energy decomposition analyses (given in percentages of: electrostatics / orbital / disposition interactions); the 'atoms-in-molecules' analyses (bond paths are this red lines and bond critical points (bcp's) are small red spheres, densities in a.u. x 100); and the non-covalent interaction (NCI) plots.

The small but positive values of the reduced density gradient projected on the noncovalent interaction isosurface indicate weak electrostatic attraction with the *sp*^{3} carbon centre (scale is from 0.25 (red) to 0.50 (blue)). The interaction energy ΔE^{BSSE} and the contribution from electrostatic attraction follow the order **10** < **11** < **12**, which is likely a reflection of the increased electron density of the lone-pair electrons on C/O/N. While this means that CF_4 can indeed act as a σ-hole donor, it must be noted that none of the bcp's involve the C-atom of **8** and that the interaction energies (ΔE^{BSSE}) are the smallest of those computed with CO, OMe_2 and NMe_3 (see Table 2, the second lowest is the C–H···C/O/N hydrogen bonding geometry with benzene ring). Given the above plus the fact that the electrostatic potential found on the MEP of **8** (+32 kcal·mol^{-1}, see Figure 1) is comparatively large (e.g. +19 kcal·mol^{-1} in benzene), it is likely that the electrostatic attraction with the positive C in CF_4 (NCI plots, energy decomposition analysis) is counteracted by the repulsion of the lone-pair electrons on F (AIM analysis).

Table S1. Numerical overview of the interaction energies (ΔE), energy decomposition analysis, and the orbitals involved in the orbital interactions between donor molecule (Me_3N, N≡C⁻, Cl⁻) and several acceptor molecules (**1**–**5**, **13**, **14**). The percentage that these orbitals contribute to the occupied molecualr orbital of the adduct is given in parantheses. See also Figures S4 for a scheme schowing the orbital interactions with **1** and **2** as acceptors (σ-holes), Figure S5 for the orbital interactions with **3**, **4a** and **4b** as acceptros (π-holes), Figure S6 for the orbital interactions with the electron deficient aromatic rings C_6F_6 (**5**) and 1,3,5-trifluoro-s-triazine (**13**, $C_3N_3F_3$), and Figure S7 for strong orbital interactions in [Cl⁻···I–Ph (**2**)]⁻ and [N≡C⁻···$C_6H_4O_2$ (**14**)]⁻.

See Figure	[donor···acceptor]	Energies in kcal·mol⁻¹ ΔE	Orbital int.	Electrostatics	Dispersion	donor orb. + acceptor orb. (% contribution to HOMO of adduct)
**S4a**	[Me_3N···H_2O (**1**)]	–9.0	–7.6 (32%)	–14.2 (59%)	–2.2 (9%)	HOMO (93.2%) + LUMO+2 (1.5%)
**S4b**	[Me_3N···I–Ph (**2**)]	–6.2	–5.5 (26%)	–11.6 (56%)	–3.7 (18%)	HOMO (53.4%) + LUMO (1.7%) &HOMO (40.2%)
**S5a**	[Me_3N···C(O)Me_2 (**3**)]	–6.1	–2.6 (18%)	–6.3 (43%)	–5.5 (38%)	HOMO (98.9%) + LUMO (0.5%)
**S5b**	[Me_3N···N(O_2)Me (**4a**)]	–6.3	–2.5 (17%)	–7.3 (50%)	–4.9 (33%)	HOMO (98.8%) + LUMO (0.6%)
**S5c**	[Me_3N···N(O_2)Ph (**4b**)]	–6.6	–3.2 (17%)	–8.1 (50%)	–7.4 (39%)	HOMO (95.9%) + LUMO (1.4%)
**S6a**	[Me_3N···C_6F_6 (**5**)]	–6.8	–2.1 (14%)	–6.3 (41%)	–7.1 (46%)	HOMO (98.0%) + HOMO-1 (0.6%)
**S6b**	[N≡C⁻···C_6F_6 (**5**)]⁻	–16.0	–8.9 (36%)	–13.6 (55%)	–2.4 (10%)	HOMO (92.2%) + LUMO+11 (2.1%)
**S6c**	[N≡C⁻···$C_3N_3F_3$ (**13**)]⁻	–19.1	–9.3 (29%)	–20.1 (63%)	–2.4 (8%)	HOMO (91.1%)+ LUMO+1 (3.8%)
**S7a**	[Cl⁻···I–Ph (**2**)]⁻	–15.8	–26.5 (52%)	–24.0 (47%)	–0.3 (0.6%)	HOMO (82.7%) + LUMO (10.0%)
**S7b**	[N≡C⁻···$C_6H_4O_2$ (**14**)]⁻	–24.2	–30.1 (59%)	–23.8 (59%)	–2.4 (4.7%)	HOMO (74.4%) + LUMO (13.8%)

It is evident from the data collected in Table 1 and Table S1 that electrostatic interactions and dispersion are always the dominant driving force behind the interaction energies (ΔE) involving trimethylamine (**12**). The total amount of attractive orbital interactions in the adducts with **12** are still substantial and range from –2.1 kcal·mol⁻¹ in [Me_3N···C_6F_6 (**5**)] to –7.6 kcal·mol⁻¹ in [Me_3N···H_2O (**1**)]. Visual inspection of the orbitals shown in Figures S4–5 make it clear that the molecular orbitals in the adducts between NMe_3 and **1**–**4** originate from the molecular orbitals of the fragments, although the contribution of the unoccupied orbital never exceeds about 2%.

Interestingly, while the interactions between trimethylamine and the π-hole bearing **3**, **4a** and **4b** are straightforward HOMO-LUMO interactions (Figure S5), hydrogen bonding with **1** and halogen bonding with **2** is more complicated (Figure S4). For [Me_3N···H_2O (**1**)], an intricate web of orbital interactions was observed and the unoccupied orbital involved in the interaction is the LUMO+2, which is further hybridized with unoccupied orbitals belonging to trimethylamine. For [Me_3N···I–Ph (**2**)], the HOMO from NMe_3 interacts with the HOMO and the LUMO of **2** to form three new orbitals. Two new occupied orbitals (HOMO and HOMO-1) consisting of the lone-pair from NMe_3 and a lone-pair from the iodide. The newly formed unoccupied orbital is the LUMO+1 and consists mostly of the σ* orbital of the C–I bond, with some contribution from the lone-pair on NMe_3 (the HOMO).

Surprisingly, for [Me_3N···C_6F_6 (**5**)] (Figure S6a) there is no orbital interaction of the donor-acceptor kind, and instead two occupied orbitals of the individual molecules mix to for two new occupied orbitals in the adduct. The contribution from orbital interactions was also lowest in this adduct (–2.1 kcal·mol⁻¹). Replacing NMe_3 with the more nucleophilic N≡C⁻ gave a complex with ΔE = –16.0 kcal·mol⁻¹ and an orbital interaction of –8.9 kcal·mol⁻¹. In the [N≡C⁻···C_6F_6 (**5**)]⁻ adducts (Figure S6b), there is a clear interaction between the cyanide lone-pair (its HOMO) and one of the unoccupied orbitals of the pentafluorophenyl ring (LUMO+11). The hybridization is evident from the resulting HOMO and the LUMO+7 in the adduct. For comparison purposes, the adducts of a cyanide anion and 1,3,5-trifluoro-s-triazine (**13**) was assessed as well (see Figure S6c). The resulting interaction energy is large (ΔE = –19.1 kcal·mol⁻¹) with a substantial contribution from orbital interactions (–9.3 kcal·mol⁻¹). With electron deficient aromatic **13**, the lone-pair (HOMO) of N≡C⁻ interacts with the LUMO+1 of **13**. This is reflected in the delocalization over both molecules of the newly formed occupied and unoccupied orbitals in the N≡C⁻ [N≡C⁻···$C_3N_3F_3$ (**13**)]⁻ adduct.

The two final adducts considered represent anionic complexes involving the σ-hole donor iodobenzene ([Cl⁻···I–Ph (**2**)]⁻) and the π-hole donor *p*-benzoquinone ([N≡C⁻···$C_6H_4O_2$ (**13**)]⁻) which are also depicted in Figure S7. In both instances, about 50% of the interaction energy can be traced back to orbital interactions. Inspection of the levels (Figure S7) reveals that there are clear donor-acceptor interactions between the HOMO of the anion and the LUMO of the σ-/π-hole donor.

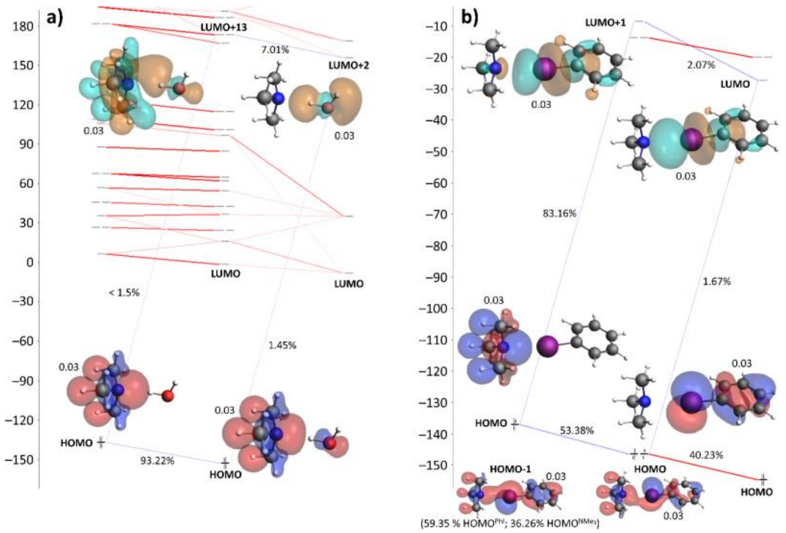

Figure S4. Selected view of energy levels of a fragment analysis conducted with ADF of adducts with NMe_3 as electron rich molecule (portrayed on the left) that is hydrogen bonded with water (**a**) or halogen bonded with iodobenzene (**b**). The levels of the [$Me_3N\cdots H_2O$] and [$Me_3N\cdots IPh$] are shown in the middle. The red and blue lines are a guide to the eye to trace the origin of the orbitals of the adduct, where blue indicates a donor-acceptor orbital interaction. The intensities of these lines are proportional to their contribution (in percentages). The portion that an orbital of a fragment contributes to the molecular orbital in the adduct are shown in percentages. Also shown are selected orbitals (at 30% opacity) that are involved in the highlighted orbital interaction. The isovalue used to generate the orbital (0.02 or 0.03) are indicted next to the orbital and the colour cade is red/blue for occupied orbitals and orange/cyan for unoccupied orbitals. The energy scale is in kcal·mol^{-1}. See also Table S1 and the accompanying small discussion.

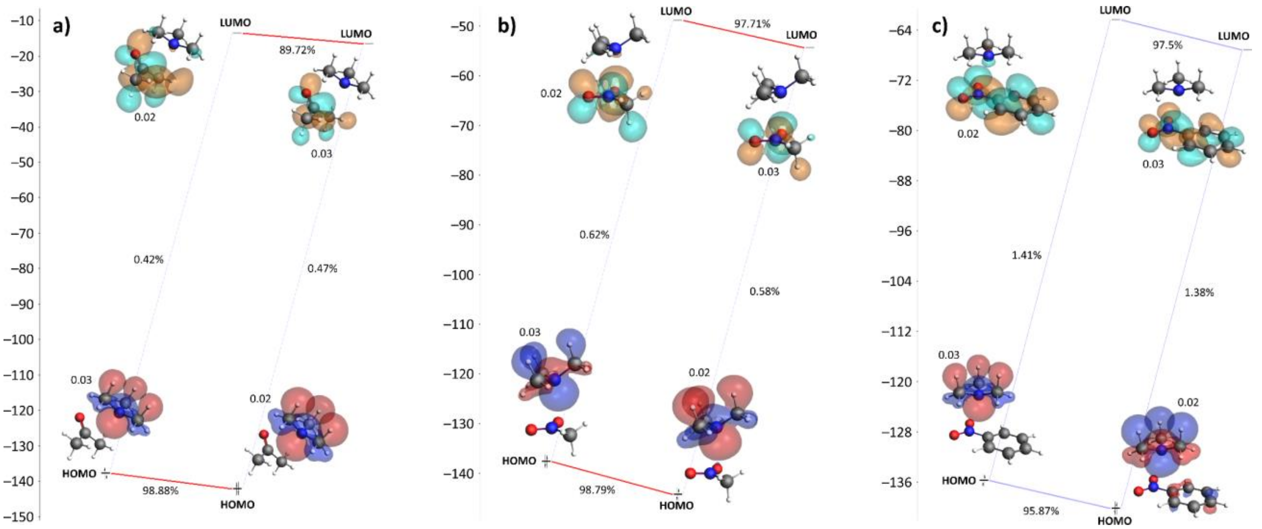

Figure S5. Selected view of energy levels of a fragment analysis conducted with ADF of adducts with trimethylamine and an electron deficient π-system in [Me_3N···acetone] (**a**), [Me_3N···nitromethane] (**b**), and [Me_3N···nitrobenzene] (**c**). The levels of the electron donor are always shown on the left, the levels of the electron poor π-molecule are portrayed on the right and the levels of the adducts are shown in the middle. The red and blue lines are a guide to the eye to trace the origin of the orbitals of the adduct, where blue indicates a donor-acceptor orbital interaction. The intensities of these lines are proportional to their contribution (in percentages) and dashed lines were not drawn by the ADF software (below the default threshold of displayed interactions) but these contributions were identified in the output file. The portion that an orbital of a fragment contributes to the molecular orbital in the adduct are shown in percentages. Also shown are selected orbitals (at 30% opacity) that are involved in the highlighted orbital interaction. The isovalue used to generate the orbital (0.02 or 0.03) are indicted next to the orbital and the colour cade is red/blue for occupied orbitals and orange/cyan for unoccupied orbitals. The energy scale is in kcal·mol^{-1}. See also Table S1 and the accompanying small discussion.

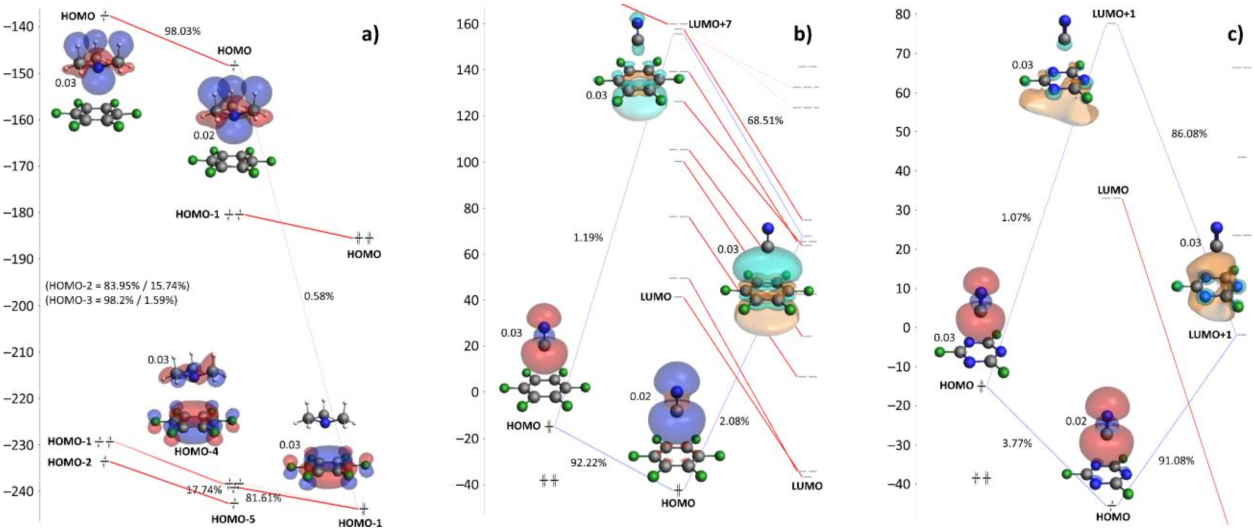

Figure S6. Selected view of energy levels of a fragment analysis conducted with ADF of adducts with an electron deficient aromatic ring in adducts [$Me_3N\cdots C_6F_6$] (**a**), [$N{\equiv}C^{-}\cdots C_6F_6$]$^-$ (**b**), and [$N{\equiv}C^{-}\cdots C_3N_3F_3$]$^-$ (**c**). The levels of the electron donor are always shown on the left, the levels of the electron poor aromatic ring are portrayed on the right and the levels of the adducts are shown in the middle. The red and blue lines are a guide to the eye to trace the origin of the orbitals of the adduct, where blue indicates a donor-acceptor orbital interaction. The intensities of these lines are proportional to their contribution (in percentages) and dashed lines were not drawn by the ADF software (below the default threshold of displayed interactions) but these contributions were identified in the output file. The portion that an orbital of a fragment contributes to the molecular orbital in the adduct are shown in percentages. Also shown are selected orbitals (at 30% opacity) that are involved in the highlighted orbital interaction. The isovalue used to generate the orbital (0.02 or 0.03) are indicted next to the orbital and the colour cade is red/blue for occupied orbitals and orange/cyan for unoccupied orbitals. The energy scale is in kcal·mol^{-1}. See also Table S1 and the accompanying small discussion.

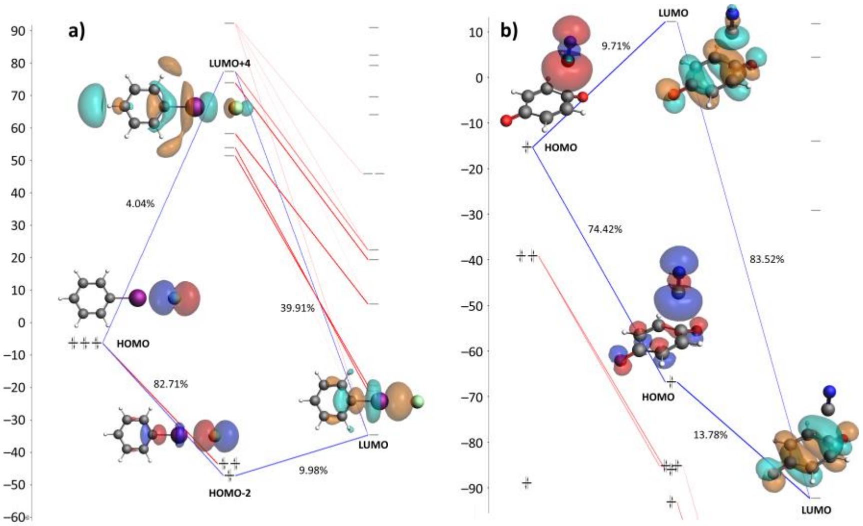

Figure S7. Selected view of energy levels of a fragment analysis conducted with ADF of adducts with the σ-hole donor iodobenzene with Cl^- (**a**) and the π-hole donor *p*-benzoquinone with ^-CN (**b**). The levels of the electron donor are always shown on the left, the levels of the σ-/π-hole are portrayed on the right and the levels of the adducts are shown in the middle. The red and blue lines are a guide to the eye to trace the origin of the orbitals of the adduct, where blue indicates a donor-acceptor orbital interaction. The portion that an orbital of a fragment contributes to the molecular orbital in the adduct are shown in percentages. Also shown are selected orbitals (at 30% opacity) that are involved in the highlighted orbital interaction. The colour code is red/blue for occupied orbitals and orange/cyan for unoccupied orbitals. The energy scale is in kcal·mol^{-1}.

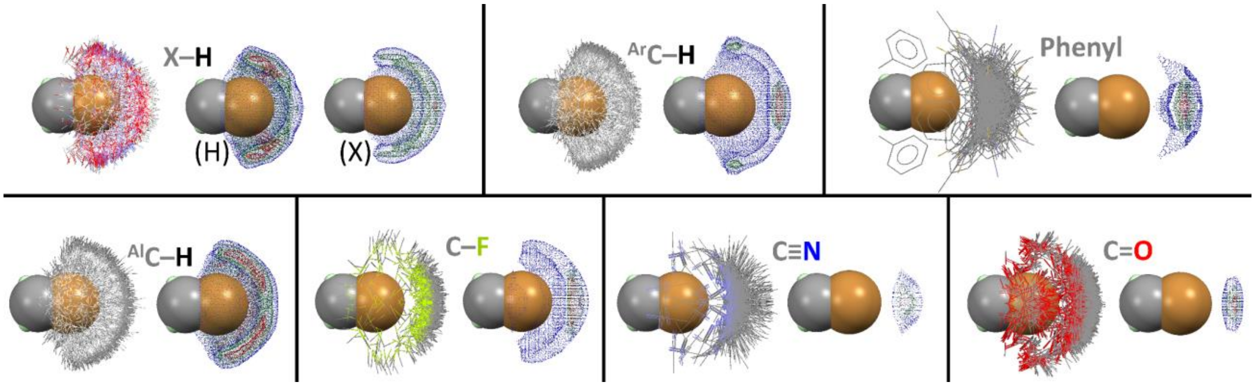

Figure S8. IsoStar data involving an aromatic bromide as 'central group' (in space filling mode). See caption of Figure 2 for general details. All the IsoStar plots were symmetry expanded.

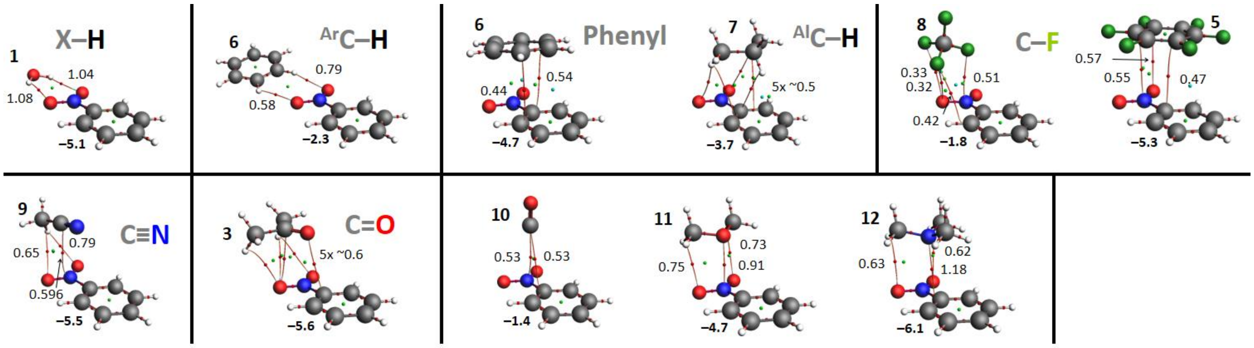

Figure S9. Model DFT calculations with nitrobenzene (**4b**) as a model for any nitro moiety as central group in the IsoStar database analysis. See caption of Figure 2 for general details. See Figure 5 for the IsoStar data involving any nitro moiety as central group and the calculations with nitromethane (**4a**) as model. Overall, the optimized structures have very similar geometries as those computed with **4a**; the O-atoms of nitrobenzene can function as hydrogen bond acceptors and the π-hole on N can act as π-hole donor. With nitrobenzene **4b**, the aryl ring sometimes participated in H–π (with **7**) or π–π interactions (with **5**). Interestingly, with water (**1**) the π–hole boning geometry is most stable for nitromethane, but not for nitrobenzene. Even starting from a π–hole boning geometry of [**1**···**4b**] resulted in convergence of the hydrogen bonded geometry shown.

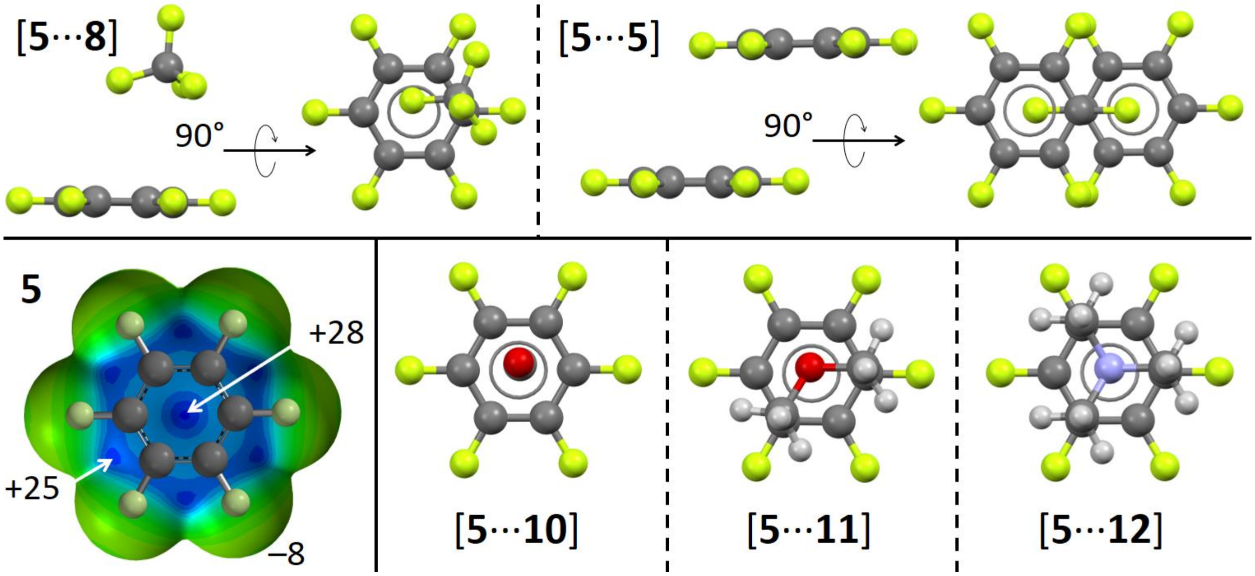

Figure S10. Alternative perspective views of DFT optimized adducts between hexafluorobenzene (**5**) and CF_4 (**8**) C_6F_6 (**5**) (top), the MEP of **5** (bottom left, see also Figure 1), and adducts of **5** with CO (**10**), OMe_2 (**11**) and NMe_3 (**12**) (bottom right). These perspectives further indicate the location of the lone-pair bearing atoms relative to the ring centre of **5**.

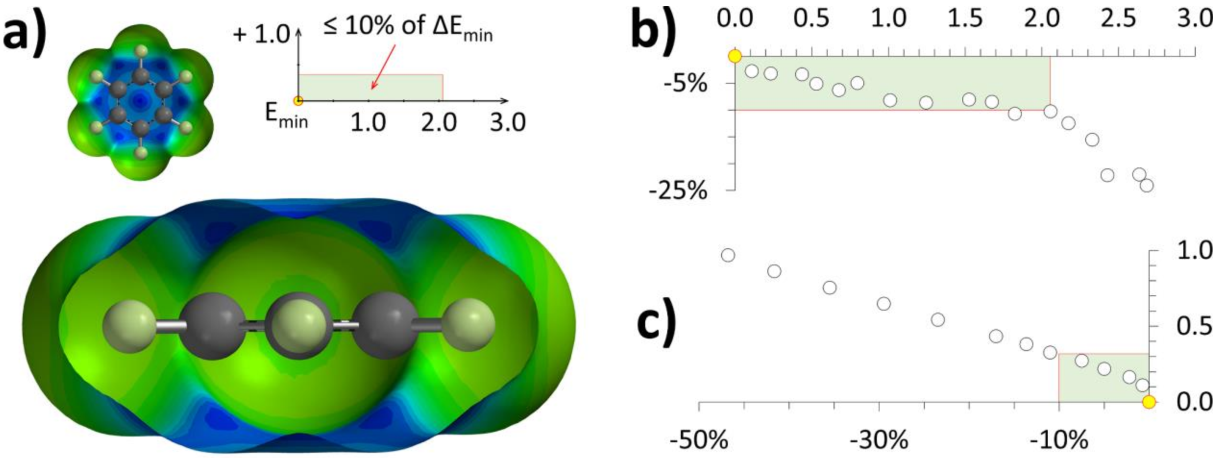

Figure S11. (**a**) Overview of the computational results of hexafluorobenzene (**5**) adducts with trimethylamine (**12**). In the unconstrained geometry optimized structure of [**5**···**12**] the N-atom of **12** is located directly above the centre of **5** (ΔE_{min} = –6.8 kcal·mol^{-1}, ADF/B3LYP-D3/TZ2P). The location of this N-atom is indicated with a yellow sphere and **5** is shown (to scale) with its MEP (ωB96X-D/6-31+G**, ± 30 kcal·mol^{-1} blue/red). The smaller MEP of **5** seen from a perspective perpendicular to the ring plane is shown as well. The inset figure represents the location of the N-atom of **12** relative to the ring centroid of **5**. This represents the translation of **12** away from the centroid perpendicular to the ring (to about 1 Å) and the parallel displacement (up to about 3 Å). The green area confined by red lines indicates the region where the computed interaction energy deviated ≤ 10% of ΔE_{min}. This figure was derived from (**b**) and (**c**), which represent the relative deviation from ΔE_{min} when displacing **12**, respectively, parallel and perpendicular to the ring plane of **5**. The data for (**b**) were obtained by constraining one F–C···N angle and allowing the adduct to geometry optimize. The data for (**c**) were obtained by constraining three C···N distances to the same value, followed by a geometry optimization.

From these data is clear that ΔE diminishes rapidly when moving trimethylamine away from the ring centroid of **5** perpendicular to the ring plane. To preserve about 90% of ΔE_{min} a movement of about 0.3 Å in this direction is tolerated. There is much more tolerance for parallel displacement, as about 90% of ΔE_{min} is preserved for a movement of about 2 Å (to either side, so a 4 Å wide patch).

Table S2. Cartesian coordinates of geometry optimized adducts (see also Table 2). The order in which adducts are provided follows the order in Table 2 from top to bottom, then left to right. The coloured filling is a guide to the eye for calculations involving the same model central group (**1**–**6**).

[1···1]				[2···12]				[4a···10]				[5···7]			
H	-0.1818	-2.1292	0.4394	C	-3.6745	2.3200	-1.4092	C	0.7697	0.7516	0.7202	C	3.8957	0.2693	-0.6490
O	0.0436	-1.3543	0.9620	C	-2.2033	0.1296	-2.2880	H	1.5207	0.3171	1.3708	C	1.4864	-0.0723	0.6909
H	-0.0007	-0.6058	0.3460	C	-4.0436	1.6632	-2.5780	H	-0.2236	0.6496	1.1534	C	2.7030	0.2422	-1.3602
H	0.8273	1.4400	-0.6874	C	-2.5735	1.8877	-0.6759	H	0.9719	1.7883	0.4720	C	3.8837	0.1251	0.7325
O	-0.0342	1.0100	-0.7223	C	-1.8409	0.7903	-1.1186	N	0.7363	-0.0353	-0.5513	C	2.6788	-0.0448	1.4025
H	-0.6542	1.6392	-0.3376	C	-3.3055	0.5689	-3.0144	O	1.0909	-1.1991	-0.4980	C	1.4986	0.0713	-0.6902
[1···6]^{HB}				N	2.2782	-0.9327	1.4524	O	0.3103	0.5306	-1.5423	F	2.7134	0.3796	-2.6848
C	0.9575	1.6417	1.0063	C	3.4276	-0.1888	0.9606	C	-2.1849	-1.1197	-0.2477	F	0.3553	0.0434	-1.3732
C	-1.1182	-0.1625	0.5913	H	3.3098	0.8699	1.1954	O	-2.8429	-1.7670	-0.8891	F	0.3311	-0.2374	1.3324
C	-0.1738	1.6859	1.8145	H	3.4957	-0.2905	-0.1233	[4a···11]				F	5.0494	0.4340	-1.2927
C	1.0504	0.6954	-0.0090	H	4.3762	-0.5389	1.4024	O	-0.8471	0.3365	-0.6298	F	5.0269	0.1508	1.4145
C	0.0128	-0.2077	-0.2176	C	2.3492	-2.3411	1.0954	C	-1.8012	-0.4858	0.0177	F	2.6661	-0.1828	2.7268
C	-1.2118	0.7836	1.6068	H	1.4354	-2.8456	1.4115	H	-2.1074	0.0249	0.9298	H	-2.8476	0.3575	2.1302
O	0.2602	-2.7194	-2.9130	H	3.2091	-2.8517	1.5614	H	-1.3711	-1.4571	0.2877	C	-2.9633	-0.2863	1.2558
H	-2.1022	0.8182	2.2426	H	-3.5860	0.0506	-3.9232	H	-2.6822	-0.6464	-0.6172	H	-2.2005	-1.0659	1.3147
H	-1.9350	-0.8728	0.4276	H	-1.6336	-0.7234	-2.6316	C	-0.3578	-0.2393	-1.8275	H	-3.9405	-0.7717	1.3240
H	-0.2472	2.4305	2.6135	H	-4.9020	2.0010	-3.1447	H	0.3482	0.4665	-2.2633	C	-2.8338	0.5025	-0.0459
H	1.7745	2.3518	1.1695	H	-4.2445	3.1729	-1.0621	H	-1.1714	-0.4206	-2.5417	H	-1.8609	1.0008	-0.0738
H	1.9405	0.6606	-0.6452	H	-2.2914	2.4009	0.2336	H	0.1645	-1.1827	-1.6308	H	-3.5866	1.2970	-0.0681
H	0.0861	-0.9535	-1.0180	H	2.4353	-2.4415	0.0127	H	0.3553	1.4504	1.4448	C	-2.9849	-0.3794	-1.2837
H	0.6742	-3.5726	-2.8441	I	-0.1552	0.1101	-0.0129	C	1.3868	1.1710	1.2657	H	-2.2227	-1.1625	-1.2990
H	0.0320	-2.5792	-3.8252	C	2.0796	-0.7431	2.8812	H	2.0014	1.3761	2.1406	H	-2.8850	0.1984	-2.2048
[1···6]$^{H\text{-}\pi}$				H	2.9279	-1.1204	3.4774	H	1.7858	1.6469	0.3782	H	-3.9625	-0.8687	-1.2990
C	1.4982	0.0927	0.2339	H	1.1773	-1.2659	3.2008	N	1.4193	-0.3095	1.0587	[5···8]			
C	-1.0867	-0.9223	0.4441	H	1.9526	0.3184	3.0973	O	2.1119	-0.7438	0.1553	C	1.3781	1.4107	-0.6081
C	0.6654	0.5159	1.2650	[3···6]^{HB}				O	0.7648	-0.9871	1.8318	C	-0.9740	0.8642	0.7643
C	1.0394	-0.8404	-0.6906	O	-1.1038	2.5925	-0.3094	[4a···12]				C	0.2060	1.1635	-1.3098
C	-0.2525	-1.3483	-0.5850	C	-0.0199	2.1780	0.0333	N	0.5245	0.0064	-0.5546	C	1.3740	1.3843	0.7807
C	-0.6273	0.0093	1.3696	C	0.4416	2.2519	1.4727	C	0.3833	0.6504	-1.8491	C	0.1980	1.1103	1.4667
O	-0.9576	1.9334	-1.6096	H	0.6858	1.2500	1.8334	H	0.2717	1.7280	-1.7165	C	-0.9696	0.8911	-0.6236
H	-1.2762	0.3425	2.1698	H	-0.3365	2.6908	2.0935	H	-0.5066	0.2731	-2.3570	F	0.2099	1.1803	-2.6415
H	-2.0941	-1.3112	0.5208	H	1.3532	2.8511	1.5440	H	1.2510	0.4756	-2.5093	F	-2.0911	0.6427	-1.2975
H	1.0225	1.2400	1.9867	C	0.9427	1.5449	-0.9438	C	1.6593	0.5342	0.1889	F	-2.0996	0.5902	1.4203
H	2.5010	0.4913	0.1483	H	1.0755	0.4911	-0.6866	H	1.5464	1.6109	0.3199	F	2.5052	1.6715	-1.2673
H	1.6860	-1.1703	-1.4941	H	1.9240	2.0221	-0.8819	H	2.6202	0.3440	-0.3200	F	2.4979	1.6219	1.4538
H	-0.6096	-2.0739	-1.3054	H	0.5520	1.6214	-1.9558	H	1.6963	0.0771	1.1778	F	0.1937	1.0794	2.7980
H	-0.7458	1.0092	-1.7824	H	-2.3780	-0.4565	-1.2183	C	0.5922	-1.4413	-0.6732	C	-0.4874	-2.7251	-0.1878
H	-0.7627	2.0321	-0.6711	C	-1.5461	-0.9795	-0.7651	H	0.6079	-1.8895	0.3212	F	-0.6852	-2.2740	1.0455
[1···7]				C	0.6263	-2.2724	0.3993	H	1.4862	-1.7824	-1.2241	F	-0.2561	-4.0281	-0.1521
C	0.0262	0.0372	-1.2993	C	-0.6956	-1.7531	-1.5476	H	-0.2882	-1.8117	-1.2034	F	0.5615	-2.1018	-0.7165
C	1.2909	-0.0540	-0.4475	C	-1.3145	-0.8571	0.6008	H	-2.2241	0.9032	0.3669	F	-1.5614	-2.4812	-0.9251
H	2.1921	-0.0610	-1.0631	C	-0.2297	-1.5044	1.1832	C	-2.2108	0.0914	1.0842	[5···5]			
H	1.2954	-0.9715	0.1487	C	0.3922	-2.3977	-0.9665	H	-2.1272	-0.8783	0.6084	C	-0.0413	-1.8550	-2.4994
H	1.3660	0.7988	0.2335	H	-0.8752	-1.8465	-2.6114	H	-3.0834	0.1323	1.7352	C	-1.4144	-1.8516	-0.0844
C	-1.2536	0.0524	-0.4657	H	-1.9729	-0.2460	1.2045	N	-1.0209	0.2791	1.9684	C	-1.4299	-1.8451	-2.4896
H	-2.1438	0.1045	-1.0950	H	-0.0503	-1.4102	2.2472	O	-0.6854	1.4219	2.2184	C	0.6611	-1.8628	-1.3015
H	-1.2736	0.9168	0.2042	H	1.0569	-2.9957	-1.5775	O	-0.4925	-0.7243	2.4179	C	-0.0258	-1.8610	-0.0942
H	-1.3337	-0.8535	0.1425	H	1.4723	-2.7747	0.8519	[4b···1]				C	-2.1166	-1.8434	-1.2824
H	0.0688	0.9404	-1.9150	[3···6]$^{H\text{-}\pi/\pi B}$				N	0.3736	-1.2665	0.3563	F	-2.1040	-1.8352	-3.6377
H	-0.0041	-0.8060	-1.9957	O	-1.8093	-0.9527	1.9048	O	1.2494	-1.8094	-0.3025	F	-3.4479	-1.8315	-1.2742
H	-0.8479	-0.0079	2.3666	C	-1.8594	-0.0540	1.0964	O	-0.2189	-1.7984	1.2850	F	-2.0724	-1.8466	1.0735
O	-0.0521	-0.0031	2.9083	C	-1.7871	1.3987	1.5141	C	0.0099	0.1180	0.0093	F	0.6156	-1.8547	-3.6571
H	0.6694	-0.0930	2.2773	H	-0.9983	1.9099	0.9583	C	-0.6741	2.7003	-0.6453	F	1.9927	-1.8673	-1.3096
[1···8]				H	-1.6010	1.4728	2.5833	C	0.6725	0.7420	-1.0394	F	0.6483	-1.8605	1.0556
F	-0.5376	-1.6339	-0.1064	H	-2.7283	1.8992	1.2705	C	-0.9872	0.7524	0.7381	C	0.0420	1.8445	0.0864
C	-0.9531	-0.5381	0.5001	C	-1.9894	-0.3138	-0.3856	C	-1.3259	2.0551	0.4019	C	1.4150	1.8619	2.4984
F	-1.1956	0.4108	-0.4014	H	-1.0773	0.0165	-0.8897	C	0.3217	2.0445	-1.3632	C	-0.6593	1.8695	1.2838
F	-2.0657	-0.7926	1.1747	H	-2.8193	0.2596	-0.8062	H	1.4393	0.2099	-1.5821	C	1.4298	1.8288	0.0942
F	-0.0186	-0.1081	1.3438	H	-2.1358	-1.3756	-0.5678	H	-1.4788	0.2280	1.5437	C	2.1164	1.8376	1.3005

H	1.0368	1.1465	-1.3348	H	2.0097	1.9490	-1.3435	H	-2.1010	2.5654	0.9580	C	0.0270	1.8780	2.4901
O	1.8313	0.6765	-1.0611	C	1.8175	0.9404	-0.9992	H	0.8259	2.5460	-2.1785	F	-1.9934	1.8745	1.2738
H	1.9025	0.8389	-0.1149	C	1.3216	-1.6503	-0.1139	H	-0.9437	3.7163	-0.9033	F	-0.6486	1.8995	3.6390
[1···5]				C	1.7344	-0.1035	-1.9153	O	0.9923	-4.6632	0.9605	F	2.0748	1.8707	3.6567
C	0.4895	0.1609	-0.8965	C	1.6557	0.6874	0.3596	H	1.4444	-4.0813	0.3390	F	-0.6207	1.8270	-1.0727
C	0.6735	-0.8251	1.6897	C	1.4067	-0.6073	0.8027	H	0.4003	-4.0589	1.4224	F	2.1022	1.8006	-1.0574
C	0.8299	-1.1666	-0.6861	C	1.4881	-1.3994	-1.4716	[4b···6]^{HB}				F	3.4494	1.8219	1.3082
C	0.2369	0.9950	0.1819	H	1.8617	0.0919	-2.9729	C	0.0135	-2.2008	1.8492	[5···9]			
C	0.3292	0.5037	1.4757	H	1.7223	1.4989	1.0736	C	0.2626	-2.8702	4.5398	C	-1.5467	1.0723	-0.4173
C	0.9232	-1.6602	0.6088	H	1.2549	-0.8021	1.8560	C	-0.1189	-3.5257	2.2530	C	-0.3873	-0.2310	1.7428
F	0.3883	0.6341	-2.1410	H	1.4202	-2.2110	-2.1853	C	0.2688	-1.2099	2.7918	C	-2.1974	0.0007	0.1780
F	-0.1082	2.2681	-0.0260	H	1.1124	-2.6546	0.2316	C	0.3942	-1.5447	4.1368	C	-0.3142	1.4884	0.0645
F	0.0837	1.3039	2.5118	[3···7]				C	0.0057	-3.8610	3.5979	C	0.2653	0.8371	1.1435
F	0.7639	-1.2980	2.9312	C	-1.6714	-0.1742	0.5391	H	-0.3176	-4.2975	1.5191	C	-1.6187	-0.6498	1.2587
F	1.2514	-2.9345	0.8118	C	-1.7033	-1.4770	-0.2575	H	-0.0763	-1.9329	0.8044	F	-2.0915	1.6850	-1.4656
F	1.0602	-1.9688	-1.7238	H	-1.5652	-2.3473	0.3883	H	-0.0969	-4.8928	3.9118	F	0.3316	2.4956	-0.5283
H	-1.9319	1.1323	-2.2433	H	-0.9166	-1.4858	-1.0148	H	0.3608	-3.1316	5.5865	F	1.4665	1.2204	1.5864
O	-2.4943	1.0019	-1.4721	H	-2.6610	-1.5934	-0.7716	H	0.5946	-0.7740	4.8712	F	0.1777	-0.8644	2.7705
H	-2.4954	1.8534	-1.0220	C	-1.8543	1.0577	-0.3460	H	0.3672	-0.1824	2.4656	F	-2.2378	-1.6836	1.8224
[1···9]				H	-1.8133	1.9820	0.2349	N	0.0411	0.6330	-1.0032	F	-3.3698	-0.4098	-0.2943
N	0.4804	-0.0594	0.0186	H	-2.8216	1.0280	-0.8539	O	0.1137	1.0632	0.1376	C	2.3601	-0.6267	-1.2346
C	-0.6613	-0.0052	-0.0873	H	-1.0791	1.1008	-1.1136	O	0.0440	-0.5529	-1.2975	H	2.5913	-0.6358	-0.1691
C	-2.1077	0.0642	-0.2195	H	-2.4478	-0.1923	1.3107	C	-0.0592	1.6128	-2.1043	H	2.3580	0.4078	-1.5809
H	-2.3758	0.6898	-1.0714	H	-0.7203	-0.1015	1.0771	C	-0.2487	3.4314	-4.1556	H	3.1352	-1.1767	-1.7688
H	-2.5443	0.4917	0.6837	O	1.2420	-0.0637	-1.6144	C	-0.0962	2.9660	-1.7974	C	1.0574	-1.2321	-1.4679
H	-2.5178	-0.9347	-0.3713	C	1.6955	0.1222	-0.5086	C	-0.1151	1.1457	-3.4104	N	0.0203	-1.6973	-1.6399
H	2.4788	0.0489	0.5069	C	2.1544	-1.0228	0.3667	C	-0.2093	2.0695	-4.4410	[5···10]			
O	3.4354	0.0662	0.6575	H	3.2306	-0.9428	0.5432	C	-0.1926	3.8780	-2.8387	C	-0.2266	-0.0347	-1.4208
H	3.8123	-0.3615	-0.1172	H	1.9328	-1.9727	-0.1142	H	-0.0507	3.2824	-0.7657	C	-0.8496	0.2971	1.2644
[1···3]				H	1.6670	-0.9766	1.3436	H	-0.0837	0.0826	-3.5976	C	-0.5964	-1.1304	-0.6540
H	-0.6551	-1.5591	-3.3572	C	1.8173	1.5110	0.0780	H	-0.2528	1.7258	-5.4665	C	-0.1688	1.2272	-0.8458
O	-0.7887	-0.7728	-2.8204	H	2.8090	1.6702	0.5074	H	-0.2250	4.9378	-2.6224	C	-0.4809	1.3940	0.4968
H	-0.7523	-1.0745	-1.8963	H	1.0933	1.6196	0.8899	H	-0.3232	4.1477	-4.9643	C	-0.9068	-0.9648	0.6885
O	-0.4710	-1.1678	-0.0043	H	1.6120	2.2588	-0.6845	[4b···6]$^{\pi B}$				F	0.0883	-0.1969	-2.7054
C	-0.0095	-0.1774	0.5263	[3···8]				N	1.9102	-1.3372	-0.9138	F	0.1914	2.2776	-1.5820
C	0.1767	-0.1150	2.0237	F	-1.8725	-1.0753	-0.4474	O	2.5115	-0.6142	-1.6929	F	-0.4282	2.6060	1.0467
H	1.1973	0.1864	2.2700	C	-1.9030	-1.5665	0.7807	O	2.4473	-2.0753	-0.1006	F	-1.1475	0.4549	2.5532
H	-0.0507	-1.0791	2.4730	F	-1.9301	-0.5674	1.6574	C	0.4376	-1.3097	-0.9487	F	-1.2513	-2.0193	1.4261
H	-0.4876	0.6473	2.4405	F	-2.9801	-2.3206	0.9373	C	-2.3132	-1.2247	-0.9755	F	-0.6337	-2.3447	-1.2011
C	0.3968	1.0446	-0.2600	F	-0.8134	-2.2993	0.9909	C	-0.1976	-0.3925	-1.7718	C	2.6592	-0.6681	0.3983
H	1.4616	1.2390	-0.1044	C	0.6650	0.8663	-0.9624	C	-0.2706	-2.1874	-0.1394	O	3.7510	-0.8980	0.5350
H	-0.1397	1.9199	0.1155	O	0.1327	1.0253	-2.0358	C	-1.6560	-2.1405	-0.1594	[5···11]			
H	0.1976	0.9110	-1.3208	C	0.3610	1.7618	0.2195	C	-1.5847	-0.3549	-1.7792	C	-0.6683	1.2237	1.6028
[1···10]				H	1.2684	2.2841	0.5341	H	0.3914	0.2803	-2.3753	C	0.7088	-1.1700	1.3509
C	-0.0919	0.8988	1.3680	H	0.0247	1.1668	1.0721	H	0.2635	-2.8798	0.4933	C	-1.3517	0.0196	1.6820
O	-0.0811	1.6709	2.1836	H	-0.4054	2.4854	-0.0491	H	-2.2236	-2.8170	0.4661	C	0.7035	1.2315	1.3976
H	0.5956	-1.3299	-1.5161	C	1.6750	-0.2368	-0.7278	H	-2.0960	0.3624	-2.4075	C	1.3928	0.0342	1.2706
O	-0.2090	-0.8049	-1.4629	H	2.6217	0.1847	-0.3798	H	-3.3952	-1.1871	-0.9820	C	-0.6625	-1.1768	1.5567
H	-0.2136	-0.4348	-0.5726	H	1.8351	-0.7974	-1.6461	H	0.9303	3.2749	-0.5449	F	-1.3339	2.3751	1.6986
[1···11]				H	1.3208	-0.9112	0.0562	C	0.7343	2.4979	0.1837	F	-2.6724	0.0119	1.8517
O	-0.2330	0.3773	0.0560	[3···5]				C	0.2297	0.4924	2.0418	F	-1.3229	-2.3345	1.5981
C	-0.3960	-0.3491	1.2613	C	-0.6737	-0.7172	2.3694	C	1.7864	1.7441	0.6949	F	1.3658	-2.3210	1.1992
H	-1.3482	-0.8726	1.1933	C	0.9649	1.0156	0.9531	C	-0.5689	2.2477	0.6003	F	2.7087	0.0405	1.0509
H	-0.4121	0.3226	2.1278	C	0.6100	-1.1086	2.0161	C	-0.8222	1.2426	1.5275	F	1.3566	2.3899	1.2982
H	0.4099	-1.0815	1.3967	C	-1.1382	0.5406	2.0137	C	1.5337	0.7432	1.6268	O	-0.6317	-0.0509	-1.3916
C	0.9859	1.0940	-0.0128	C	-0.3182	1.4071	1.3056	H	2.7984	1.9242	0.3557	C	-0.0575	1.1401	-1.8882
H	1.0099	1.5983	-0.9774	C	1.4297	-0.2420	1.3085	H	-1.3883	2.8281	0.1945	H	-0.5628	1.9733	-1.4007
H	1.8499	0.4217	0.0653	F	-1.4650	-1.5548	3.0333	H	-1.8380	1.0363	1.8407	H	-0.1883	1.2299	-2.9742
H	1.0495	1.8424	0.7865	F	1.0488	-2.3237	2.3383	H	2.3490	0.1442	2.0116	H	1.0158	1.1932	-1.6607
H	-0.6391	-1.7471	-2.3086	F	2.6526	-0.6275	0.9402	H	0.0311	-0.2982	2.7542	C	-0.0588	-1.2141	-1.9540
O	-1.3187	-1.0866	-2.1448	F	1.7409	1.8353	0.2377	[4b···7]				H	-0.5715	-2.0722	-1.5209
H	-0.9580	-0.5194	-1.4432	F	-0.7742	2.6036	0.9314	N	1.7975	-1.2708	-1.1365	H	1.0126	-1.2861	-1.7230
[1···12]				F	-2.3766	0.9083	2.3323	O	2.3821	-0.5798	-1.9569	H	-0.1824	-1.2370	-3.0442
N	-0.0021	0.0199	-0.1202	O	-1.2206	-1.0653	-0.7408	O	2.3465	-2.0336	-0.3564	[5···12]			

C	-0.7523	-1.1850	-0.4577	C	-0.5385	-0.4896	-1.5592	C	0.3268	-1.1662	-1.0745	C	-1.4457	1.2546	-1.2514
H	-1.7166	-1.1696	0.0508	C	-0.9587	0.8388	-2.1474	C	-2.4106	-0.9256	-0.9167	C	-2.0418	0.9660	1.4481
H	-0.2053	-2.0641	-0.1164	H	-0.2705	1.6196	-1.8108	C	-0.3193	-0.2767	-1.9212	C	-1.7733	0.0038	-0.7419
H	-0.9281	-1.2793	-1.5415	H	-1.9684	1.0881	-1.8290	C	-0.3639	-1.9418	-0.1541	C	-1.4166	2.3612	-0.4111
C	-0.7324	1.2275	-0.4831	H	-0.9023	0.8171	-3.2380	C	-1.7436	-1.8154	-0.0805	C	-1.7142	2.2169	0.9387
H	-1.6981	1.2392	0.0235	C	0.7903	-1.0429	-2.0209	C	-1.6992	-0.1602	-1.8356	C	-2.0710	-0.1406	0.6079
H	-0.9090	1.3025	-1.5687	H	1.5878	-0.3267	-1.8064	H	0.2572	0.3122	-2.6181	F	-1.1635	1.3947	-2.5543
H	-0.1708	2.1081	-0.1674	H	0.7809	-1.1889	-3.1039	H	0.1777	-2.6170	0.4908	F	-1.1048	3.5685	-0.9034
C	1.3362	0.0006	-0.6956	H	0.9989	-1.9870	-1.5232	H	-2.2973	-2.4088	0.6350	F	-1.6911	3.2852	1.7481
H	1.8882	0.8868	-0.3785	[3···9]				H	-2.2181	0.5340	-2.4832	F	-2.3332	0.8275	2.7492
H	1.3200	-0.0196	-1.7977	N	-0.8476	-1.5074	1.3264	H	-3.4865	-0.8261	-0.8502	F	-2.3919	-1.3462	1.0983
H	1.8744	-0.8799	-0.3427	C	-0.0298	-0.9203	1.8822	C	0.0475	1.4349	1.3337	F	-1.8056	-1.0629	-1.5532
H	0.0944	-0.1542	1.7453	C	1.0089	-0.1574	2.5545	H	-0.7002	0.8624	0.7790	N	1.3831	0.5487	0.7285
O	0.0707	-0.3752	2.7025	H	1.8953	-0.7765	2.6944	H	-0.5120	2.1287	1.9688	C	2.1721	1.7130	0.3137
H	0.5308	0.3422	3.1472	H	0.6566	0.1813	3.5295	C	0.8637	0.4884	2.2107	H	2.0330	1.9087	-0.7554
[2···1]^{HB}				H	1.2690	0.7038	1.9377	H	1.5743	1.0406	2.8312	H	3.2433	1.5790	0.5039
C	-0.7500	0.0000	-0.6121	O	0.8877	1.1720	-0.3862	H	1.4391	-0.2173	1.6068	H	1.8369	2.6114	0.8439
C	-0.7065	0.0000	2.1553	C	0.0589	0.5799	-1.0450	H	0.2220	-0.0936	2.8760	C	1.5487	0.3218	2.1677
C	0.4750	0.0000	0.0455	C	0.4138	-0.6444	-1.8530	C	0.9093	2.2285	0.3532	H	1.2009	1.1922	2.7351
C	-1.9503	0.0000	0.0886	H	-0.1409	-1.5003	-1.4603	H	1.6298	2.8575	0.8825	H	2.5919	0.1256	2.4408
C	-1.9207	0.0000	1.4790	H	1.4821	-0.8392	-1.7898	H	0.3003	2.8790	-0.2787	H	0.9404	-0.5294	2.4936
C	0.4837	0.0000	1.4375	H	0.1184	-0.5148	-2.8970	H	1.4768	1.5668	-0.3052	C	1.8164	-0.6378	-0.0161
H	1.4067	0.0000	-0.5031	C	-1.3892	1.0042	-1.0687	[4b···8]				H	1.6701	-0.4893	-1.0918
H	-2.8966	0.0000	-0.4352	H	-1.9881	0.2209	-0.5969	N	1.8416	-0.6987	-0.2571	H	1.2135	-1.5082	0.2659
H	-2.8536	0.0000	2.0286	H	-1.7459	1.1153	-2.0952	O	2.4010	0.2493	-0.7883	H	2.8717	-0.8769	0.1593
H	1.4338	0.0000	1.9564	H	-1.5170	1.9383	-0.5257	O	2.4039	-1.5399	0.4252	[6···6]^{HB}			
H	-0.6890	0.0000	3.2374	[3···3]				C	0.3835	-0.8290	-0.4494	H	-1.3333	-2.4978	1.9926
I	-0.8122	0.0000	-2.7369	O	0.9012	0.0104	1.4622	C	-2.3372	-1.0590	-0.7898	C	-0.3285	-2.2005	1.7193
H	3.6966	0.0000	-2.8999	C	1.5376	0.0045	0.4291	C	-0.2689	0.0788	-1.2721	C	2.2494	-1.4301	1.0076
O	2.9239	0.0000	-2.3261	C	1.9260	-1.2826	-0.2563	C	-0.2912	-1.8487	0.2075	C	0.2913	-1.1465	2.3826
H	2.1592	0.0000	-2.9149	H	1.3821	-1.3457	-1.2026	C	-1.6636	-1.9576	0.0319	C	0.3412	-2.8695	0.6995
[2···1]^{Hlg}				H	1.6674	-2.1347	0.3686	C	-1.6409	-0.0448	-1.4405	C	1.6302	-2.4848	0.3445
C	-0.0492	1.1895	-0.6921	H	2.9931	-1.2951	-0.4890	H	0.2942	0.8609	-1.7575	C	1.5792	-0.7609	2.0262
C	-0.0593	-1.1997	-2.0856	C	1.9332	1.2837	-0.2668	H	0.2560	-2.5316	0.8396	H	-0.2313	-0.6216	3.1724
C	-0.0583	1.2010	-2.0804	H	1.3808	1.3461	-1.2083	H	-2.2072	-2.7446	0.5377	H	-0.1427	-3.6860	0.1779
C	-0.0459	-0.0032	0.0225	H	2.9979	1.2843	-0.5097	H	-2.1668	0.6530	-2.0787	H	2.1490	-3.0043	-0.4516
C	-0.0501	-1.1918	-0.6979	H	1.6883	2.1418	0.3554	H	-3.4079	-1.1489	-0.9229	H	2.0567	0.0651	2.5378
C	-0.0638	0.0017	-2.7795	O	-0.8941	0.0054	-1.4577	C	1.2850	2.5125	1.5412	H	3.2505	-1.1272	0.7278
F	-0.0443	2.3670	-0.0603	C	-1.5357	0.0012	-0.4279	F	1.0743	1.2561	1.9196	H	-2.9531	2.8439	-0.3196
F	-0.0618	2.3553	-2.7498	C	-1.9284	-1.2850	0.2568	F	2.5751	2.7042	1.3296	C	-2.0802	2.3929	-0.7757
F	-0.0725	0.0031	-4.1116	H	-1.3780	-1.3534	1.1992	F	0.8635	3.3326	2.4974	C	0.1607	1.2350	-1.9465
F	-0.0637	-2.3531	-2.7572	H	-1.6801	-2.1371	-0.3724	F	0.6055	2.7552	0.4264	C	-1.4730	3.0041	-1.8683
F	-0.0463	-2.3727	-0.0712	H	-2.9937	-1.2914	0.4974	[4b···5]				C	-1.5664	1.2031	-0.2700
I	-0.0508	-0.0160	2.1198	C	-1.9320	1.2814	0.2656	C	-0.4513	2.4343	-1.8380	C	-0.4461	0.6225	-0.8552
O	-0.0557	0.0016	5.0450	H	-1.3858	1.3424	1.2109	C	-1.3673	1.1719	0.4589	C	-0.3518	2.4249	-2.4537
H	0.3638	-0.7594	5.4603	H	-2.9985	1.2850	0.5010	C	-1.4099	1.4337	-1.9310	H	-1.8734	3.9298	-2.2631
H	0.3580	0.7768	5.4379	H	-1.6812	2.1388	-0.3553	C	0.0498	2.8020	-0.5966	H	-2.0366	0.7256	0.5809
[2···6]$^{\pi B}$				[3···10]				C	-0.4065	2.1688	0.5512	H	-0.0477	-0.3015	-0.4602
I	-2.9172	-1.4932	1.0868	O	-0.7747	-0.4718	-1.3990	C	-1.8659	0.8008	-0.7820	H	0.1216	2.9006	-3.3040
C	-1.0189	-1.6992	0.1697	C	0.1275	-0.1505	-0.6599	F	-1.8818	1.0729	-3.1201	H	1.0341	0.7833	-2.4012
C	1.4529	-1.9692	-1.0522	C	0.8735	-1.1668	0.1751	F	-0.0075	3.0335	-2.9385	[6···6]$^{\pi B}$			
C	-0.9507	-1.8662	-1.2080	H	1.9529	-1.0666	0.0373	F	0.9796	3.7510	-0.5082	H	-1.5582	0.9252	2.2528
C	0.1325	-1.6561	0.9451	H	0.5541	-2.1729	-0.0874	F	0.0909	2.5047	1.7422	C	-1.1761	-0.0083	1.8589
C	1.3690	-1.7947	0.3235	H	0.6658	-0.9824	1.2329	F	-1.7950	0.5518	1.5588	C	-0.1747	-2.3898	0.8320
C	0.2920	-2.0010	-1.8153	C	0.5552	1.2915	-0.5025	F	-2.7689	-0.1757	-0.8684	C	-0.1801	-0.6958	2.5435
H	-1.8524	-1.8832	-1.8049	H	1.6024	1.4066	-0.7943	N	0.9199	-0.9283	-1.6460	C	-1.6705	-0.5097	0.6601
H	0.0695	-1.5072	2.0134	H	0.4802	1.5886	0.5466	O	1.6576	0.0045	-1.9315	C	-1.1705	-1.7010	0.1482
H	2.2690	-1.7556	0.9232	H	-0.0707	1.9366	-1.1149	O	0.2177	-1.5246	-2.4470	C	0.3217	-1.8873	2.0300
H	0.3463	-2.1264	-2.8897	O	-3.5240	-0.2102	1.1712	C	0.8516	-1.3404	-0.2308	H	0.2063	-0.3023	3.4756
H	2.4183	-2.0744	-1.5298	C	-2.4422	-0.0019	1.3949	C	0.6435	-2.0290	2.4222	H	-2.4321	0.0348	0.1172
H	-2.4571	2.0780	1.2063	[3···11]				C	-0.0567	-2.3231	0.1367	H	-1.5457	-2.0846	-0.7922
C	-1.4390	1.9742	0.8555	H	0.7970	-1.3242	2.6608	C	1.6658	-0.7003	0.6934	H	1.0986	-2.4228	2.5616
C	1.1721	1.6766	-0.0522	C	0.4042	-1.2810	1.6417	C	1.5553	-1.0529	2.0303	H	0.2176	-3.3150	0.4278
C	-1.1914	1.7602	-0.4946	H	-0.2382	-2.1393	1.4584	C	-0.1570	-2.6634	1.4782	H	-0.2004	0.3070	-3.4795

C	-0.3803	2.0387	1.7547
C	0.9257	1.8921	1.2995
C	0.1129	1.6092	-0.9499
H	-2.0202	1.6982	-1.1865
H	-0.5728	2.1965	2.8086
H	1.7504	1.9340	2.0001
H	0.3016	1.4230	-1.9993
H	2.1876	1.5458	-0.4039
[2···6]$^{\mathrm{Hlg}\text{-}\pi}$			
C	-3.4349	2.4749	-1.5830
C	-2.4611	-0.1199	-1.8244
C	-4.0120	1.6016	-2.4982
C	-2.3724	2.0610	-0.7851
C	-1.8927	0.7615	-0.9113
C	-3.5228	0.3059	-2.6159
H	-3.9649	-0.3818	-3.3262
H	-2.0839	-1.1289	-1.9195
H	-4.8386	1.9285	-3.1161
H	-3.8103	3.4859	-1.4856
H	-1.9268	2.7436	-0.0751
I	-0.2721	0.1107	0.2822
H	3.8887	-1.8773	-0.4862
C	3.4527	-1.5627	0.4536
C	2.3193	-0.7471	2.8606
C	2.6595	-2.4428	1.1824
C	3.6791	-0.2746	0.9277
C	3.1130	0.1334	2.1308
C	2.0919	-2.0348	2.3853
H	2.4762	-3.4426	0.8093
H	4.2887	0.4133	0.3553
H	3.2824	1.1383	2.4962
H	1.4677	-2.7172	2.9485
H	1.8733	-0.4290	3.7946
[2···7]			
I	-0.7846	2.9742	1.4362
C	-1.3848	1.3183	0.2632
C	-2.0734	-0.8833	-1.2743
C	-1.1820	1.3530	-1.1108
C	-1.9331	0.2008	0.8792
C	-2.2750	-0.9008	0.1009
C	-1.5300	0.2453	-1.8767
H	-0.7476	2.2232	-1.5813
H	-2.0814	0.1767	1.9491
H	-2.6930	-1.7785	0.5777
H	-1.3643	0.2651	-2.9466
H	-2.3310	-1.7481	-1.8725
H	2.4071	-0.7724	2.1667
C	1.4623	-0.8168	1.6185
H	0.8548	0.0315	1.9404
H	0.9438	-1.7308	1.9156
C	1.6948	-0.7702	0.1097
H	0.7377	-0.8836	-0.4057
H	2.3098	-1.6239	-0.1921
C	2.3635	0.5269	-0.3404
H	1.7513	1.3911	-0.0742
H	2.5173	0.5466	-1.4215
H	3.3378	0.6558	0.1390
[2···8]			
H	0.7201	-1.6631	-3.7036
C	0.7567	-0.8188	-3.0267
C	0.8379	1.3456	-1.2765
C	-0.0035	-0.8463	-1.8618
C	1.5536	0.2811	-3.3219
C	1.5916	1.3598	-2.4458

H	1.2575	-1.3009	0.9609
C	-0.3670	0.0023	1.4326
C	0.4064	1.2837	1.6457
H	-0.2369	2.1440	1.4751
H	1.2536	1.3084	0.9576
H	0.8084	1.3183	2.6616
O	-1.5348	0.0038	1.1128
O	0.4625	-0.0011	-1.4088
C	-0.2448	-1.1768	-1.7583
H	-0.4346	-1.2189	-2.8389
H	-1.2004	-1.2368	-1.2239
H	0.3769	-2.0263	-1.4772
C	-0.2463	1.1735	-1.7587
H	0.3736	2.0238	-1.4763
H	-1.2027	1.2317	-1.2257
H	-0.4345	1.2159	-2.8396
[3···12]			
N	-0.4763	-0.6133	-0.7828
C	0.2796	-0.0932	-1.9116
H	1.3310	-0.0051	-1.6372
H	-0.0887	0.8995	-2.1777
H	0.2019	-0.7377	-2.8048
C	0.0408	-1.9008	-0.3469
H	1.0898	-1.7963	-0.0699
H	-0.0428	-2.6768	-1.1282
H	-0.5129	-2.2450	0.5288
C	-1.8997	-0.6696	-1.0655
H	-2.4425	-1.0024	-0.1784
H	-2.1454	-1.3596	-1.8927
H	-2.2656	0.3228	-1.3368
C	1.2187	0.9166	1.2330
O	2.2281	0.4201	0.7849
C	0.6697	2.2218	0.7046
H	0.7304	2.9896	1.4813
H	-0.3819	2.1022	0.4407
H	1.2396	2.5409	-0.1649
C	0.4541	0.2821	2.3728
H	0.9419	-0.6393	2.6826
H	-0.5668	0.0711	2.0499
H	0.3968	0.9723	3.2189
[4a···1]^{HB}			
O	-0.0004	0.0146	3.1567
H	-0.9046	-0.5034	-2.1826
C	0.0000	-0.0107	-1.8425
H	-0.0017	1.0372	-2.1374
H	0.9052	-0.5011	-2.1842
N	0.0011	-0.0253	-0.3455
O	-1.0820	-0.0144	0.2130
O	1.0850	-0.0171	0.2114
H	0.7521	0.0092	2.5544
H	-0.7547	0.0109	2.5567
[4a···1]$^{\pi\mathrm{B/HB}}$			
O	1.3889	0.9647	-1.3073
H	-1.0822	0.3585	1.7868
C	-1.0966	0.2137	0.7118
H	-0.7202	1.0941	0.1926
H	-2.0729	-0.0723	0.3349
N	-0.1286	-0.8720	0.3728
O	-0.3407	-1.5152	-0.6412
O	0.8411	-1.0062	1.0977
H	2.0623	0.6331	-0.7024
H	1.1489	0.2016	-1.8458
[4a···6]^{HB}			
C	-0.0645	-1.0913	0.4038

H	-0.6757	-2.7880	-0.6160
H	2.3545	0.0616	0.3606
H	2.1745	-0.5585	2.7666
H	-0.8697	-3.4167	1.7865
H	0.5525	-2.2906	3.4685
[4b···9]			
N	1.4526	-0.2285	-0.1180
O	1.9563	0.6861	-0.7551
O	1.9711	-0.7389	0.8665
C	0.1406	-0.7308	-0.5474
C	-2.3292	-1.6494	-1.3349
C	-0.5123	-0.0898	-1.5907
C	-0.4130	-1.8178	0.1150
C	-1.6574	-2.2772	-0.2902
C	-1.7577	-0.5578	-1.9813
H	-0.0521	0.7614	-2.0686
H	0.1245	-2.2792	0.9294
H	-2.1057	-3.1230	0.2144
H	-2.2858	-0.0653	-2.7871
H	-3.3032	-2.0088	-1.6420
N	-0.5714	1.8905	1.4915
C	0.5307	2.1549	1.6861
C	1.9341	2.4620	1.9134
H	2.5280	1.5559	1.7881
H	2.0754	2.8534	2.9212
H	2.2743	3.2025	1.1895
[4b···3]			
N	1.2145	-1.1146	-0.1835
O	1.9321	-0.2593	-0.6890
O	1.5655	-1.8428	0.7316
C	-0.1533	-1.2595	-0.7017
C	-2.7234	-1.5070	-1.6453
C	-0.5786	-0.4152	-1.7174
C	-0.9820	-2.2225	-0.1435
C	-2.2769	-2.3429	-0.6261
C	-1.8761	-0.5462	-2.1885
H	0.0956	0.3279	-2.1141
H	-0.6120	-2.8493	0.6533
H	-2.9398	-3.0857	-0.2025
H	-2.2289	0.1061	-2.9763
H	-3.7368	-1.6020	-2.0144
C	0.7285	1.6533	1.3479
C	1.7019	1.1103	2.3671
H	2.4559	0.5151	1.8446
H	1.1820	0.4731	3.0787
H	2.2232	1.9175	2.8859
C	1.2456	2.7370	0.4299
H	2.0964	2.3470	-0.1336
H	1.6006	3.5931	1.0088
H	0.4623	3.0526	-0.2560
O	-0.3962	1.2138	1.2443
[4b···10]			
N	2.1381	0.0277	0.0882
O	2.7037	0.8249	-0.6433
O	2.6666	-0.5464	1.0272
C	0.7140	-0.2565	-0.1767
C	-1.9514	-0.7608	-0.6488
C	0.0730	0.4230	-1.2028
C	0.0547	-1.1844	0.6172
C	-1.2884	-1.4346	0.3725
C	-1.2710	0.1651	-1.4338
H	0.6237	1.1377	-1.7959
H	0.5918	-1.6886	1.4064
H	-1.8183	-2.1555	0.9814

C	0.1838	0.6989	-2.5458
C	1.1672	1.6984	-0.1455
C	1.1801	0.0116	-1.8616
C	-0.3213	1.8879	-2.0299
C	0.1717	2.3876	-0.8295
C	1.6710	0.5098	-0.6602
H	1.5647	-0.9199	-2.2580
H	-1.0988	2.4224	-2.5616
H	-0.2243	3.3103	-0.4234
H	2.4318	-0.0360	-0.1178
H	1.5386	2.0787	0.7975
[6···7]			
C	-1.7623	1.8390	-0.5790
C	-1.4852	-0.9236	-0.7593
C	-1.1958	1.2688	-1.7143
C	-2.1889	1.0269	0.4671
C	-2.0511	-0.3541	0.3764
C	-1.0571	-0.1123	-1.8041
C	1.6758	0.1133	0.7377
C	1.3030	-0.8191	1.8880
H	0.8225	-0.2759	2.7045
H	2.1846	-1.3234	2.2927
H	0.6061	-1.5888	1.5472
C	2.3075	-0.6319	-0.4362
H	2.5599	0.0457	-1.2547
H	1.6205	-1.3852	-0.8293
H	-0.6084	-0.5551	-2.6846
H	-1.3700	-1.9982	-0.8266
H	-0.8591	1.8996	-2.5275
H	-1.8683	2.9143	-0.5085
H	-2.6268	1.4698	1.3529
H	3.2237	-1.1446	-0.1303
H	0.7807	0.6378	0.3971
H	2.3671	0.8821	1.0976
H	-2.3782	-0.9852	1.1931
[6···8]			
H	1.5901	-1.7994	-1.7580
C	1.5681	-1.1059	-0.9265
C	1.5111	0.6751	1.2096
C	0.3492	-0.6867	-0.4031
C	2.7584	-0.6350	-0.3821
C	2.7299	0.2554	0.6865
C	0.3206	0.2035	0.6656
H	-0.5774	-1.0544	-0.8248
H	3.7069	-0.9628	-0.7892
H	3.6565	0.6223	1.1101
H	1.4885	1.3684	2.0414
F	-3.1144	-0.6659	-0.4874
C	-3.6958	0.5192	-0.3415
F	-3.5112	1.2395	-1.4382
F	-4.9941	0.3504	-0.1329
F	-3.1583	1.1474	0.6973
H	-0.6280	0.5289	1.0733
[6···9]			
H	2.1649	-2.1873	-1.3817
C	2.2057	-1.2977	-0.7647
C	2.3126	0.9843	0.8207
C	1.0307	-0.6427	-0.4101
C	3.4347	-0.8127	-0.3269
C	3.4883	0.3289	0.4662
C	1.0844	0.4988	0.3831
H	0.0689	-1.0099	-0.7433
H	4.3487	-1.3240	-0.6037
H	4.4444	0.7067	0.8075

C	0.0428	0.2399	-0.9962	C	0.1650	-1.7047	3.1094	H	-1.7877	0.6885	-2.2278	H	0.1631	0.9984	0.6528
H	-0.6256	-1.7020	-1.6378	C	-0.2165	-2.4053	0.8353	H	-3.0000	-0.9574	-0.8333	H	2.3546	1.8737	1.4378
H	2.1401	0.2980	-4.2315	C	0.2017	-0.0831	1.3260	O	0.6192	3.2815	2.5708	N	-2.4120	0.1141	-0.1192
H	2.2091	2.2208	-2.6688	C	0.3169	-0.3903	2.6784	C	0.9320	2.4358	1.8986	C	-3.5555	0.2227	-0.0849
H	0.8696	2.1864	-0.5982	C	-0.1014	-2.7128	2.1875	[4b⋯11]				C	-5.0034	0.3601	-0.0418
F	-2.3531	0.0994	4.0356	H	-0.4234	-3.1900	0.1178	N	-0.6218	-1.5420	-0.4823	H	-5.3527	0.9236	-0.9076
I	-1.1184	0.2171	0.7754	H	-0.1506	-0.8431	-0.6460	O	0.2159	-2.1041	-1.1716	H	-5.3034	0.8865	0.8648
C	-1.7932	-0.6249	4.9992	H	-0.2187	-3.7360	2.5232	O	-1.2118	-2.0689	0.4493	H	-5.4740	-0.6235	-0.0491
F	-2.4639	-1.7601	5.1417	H	0.2548	-1.9442	4.1619	C	-0.9520	-0.1425	-0.8039	[6⋯10]			
F	-0.5350	-0.8895	4.6799	H	0.5248	0.3937	3.3961	C	-1.5848	2.4642	-1.4204	C	-1.0486	1.8214	0.2060
F	-1.8288	0.0566	6.1369	H	0.3182	0.9352	0.9787	C	-0.2746	0.4862	-1.8378	C	-0.0125	-0.7604	0.2292
[2⋯5]				N	-0.0143	1.8389	-2.3890	C	-1.9375	0.5003	-0.0701	C	-1.7231	0.8072	0.8785
C	1.2747	-1.7643	0.4788	O	0.2292	2.2691	-1.2759	C	-2.2521	1.8141	-0.3868	C	0.1439	1.5449	-0.4552
C	2.3137	-2.0727	3.0279	O	-0.2083	0.6701	-2.6729	C	-0.5975	1.8005	-2.1424	C	0.6620	0.2537	-0.4427
C	0.6303	-2.6011	1.3815	C	-0.1214	2.8362	-3.5028	H	0.4854	-0.0526	-2.3830	C	-1.2047	-0.4839	0.8905
C	2.4304	-1.0774	0.8336	H	0.4713	3.7046	-3.2378	H	-2.4392	-0.0280	0.7264	H	-1.7304	-1.2739	1.4127
C	2.9466	-1.2378	2.1145	H	-1.1752	3.0984	-3.5778	H	-3.0207	2.3291	0.1742	H	0.3952	-1.7632	0.2338
C	1.1583	-2.7512	2.6587	H	0.2123	2.3548	-4.4159	H	-0.0785	2.3054	-2.9464	H	-2.6522	1.0228	1.3918
H	-0.2682	-3.1309	1.0967	[4a⋯6]$^{\pi B}$				H	-1.8350	3.4886	-1.6642	H	-1.4535	2.8260	0.1959
H	2.9233	-0.4279	0.1236	H	-1.3982	0.3026	0.1298	O	1.4218	-0.3707	1.2104	H	0.6681	2.3347	-0.9790
H	3.8476	-0.7064	2.3931	C	-2.0082	-0.1314	0.9181	C	2.5433	-1.0210	0.6462	H	1.5887	0.0318	-0.9568
H	0.6597	-3.4044	3.3636	H	-2.2060	-1.1812	0.7370	H	3.4034	-0.9949	1.3283	C	2.8016	-2.8059	-1.1297
H	2.7196	-2.1953	4.0234	H	-2.9180	0.4485	1.0482	H	2.7978	-0.4886	-0.2693	O	3.5657	-3.5552	-1.4750
I	0.4840	-1.5241	-1.4745	N	-1.2201	-0.0092	2.1833	H	2.3163	-2.0641	0.3954	[6⋯11]			
C	-2.8642	1.3214	-1.0637	O	-0.9987	1.1205	2.5818	C	0.9981	-0.9671	2.4213	C	0.6208	0.6817	-2.1703
C	-0.6608	2.4994	-2.2773	O	-0.8395	-1.0344	2.7203	H	1.7875	-0.9263	3.1839	C	1.1233	-1.9523	-2.9077
C	-2.3977	0.8379	-2.2772	H	0.0467	-0.2996	-3.1324	H	0.7020	-2.0121	2.2692	C	1.0540	0.3916	-3.4604
C	-2.2294	2.3964	-0.4571	C	0.4497	-0.1510	-2.1382	H	0.1340	-0.4056	2.7737	C	0.4369	-0.3419	-1.2450
C	-1.1278	2.9851	-1.0639	C	1.4894	0.2314	0.4146	[4b⋯12]				C	0.6898	-1.6588	-1.6186
C	-1.2971	1.4267	-2.8833	C	0.7732	-1.2490	-1.3469	N	1.7326	-0.9705	-0.8178	C	1.3058	-0.9262	-3.8294
F	-3.9159	0.7507	-0.4784	C	0.6453	1.1388	-1.6525	O	2.2880	-0.1855	-1.5711	N	-0.6568	0.4574	1.9250
F	-2.6792	2.8654	0.7058	C	1.1653	1.3299	-0.3757	O	2.2960	-1.5856	0.0748	C	-0.1612	1.8032	2.1556
F	-0.5194	4.0176	-0.4821	C	1.2925	-1.0576	-0.0703	C	0.2894	-1.2052	-1.0100	H	-0.5458	2.4774	1.3882
F	0.4022	3.0565	-2.8545	H	0.6191	-2.2523	-1.7244	C	-2.4145	-1.6175	-1.3490	H	0.9286	1.8118	2.0983
F	-0.8369	0.9412	-4.0364	H	0.3920	1.9929	-2.2682	C	-0.3768	-0.5136	-2.0107	H	-0.4567	2.2036	3.1419
F	-2.9939	-0.2047	-2.8526	H	1.3084	2.3318	0.0082	C	-0.3627	-2.1065	-0.1818	C	-0.1056	-0.4924	2.8752
[2⋯9]				H	1.5324	-1.9098	0.5522	C	-1.7248	-2.3085	-0.3574	H	-0.4408	-1.5011	2.6277
I	-0.2731	-0.2882	0.3594	H	1.8748	0.3792	1.4151	C	-1.7387	-0.7237	-2.1740	H	-0.4053	-0.2778	3.9168
H	0.8429	-1.3459	-4.6033	[4a⋯7]				H	0.1680	0.1798	-2.6332	H	1.6419	-1.1534	-4.8337
C	0.9364	-0.5985	-3.8250	C	-1.3309	0.1411	0.5460	H	0.1929	-2.6235	0.5857	H	1.3191	-2.9784	-3.1946
C	1.1677	1.3091	-1.8137	C	-1.1223	-1.3204	0.1494	H	-2.2487	-3.0060	0.2831	H	1.1958	1.1901	-4.1784
C	0.4131	-0.8591	-2.5628	H	-0.8003	-1.9283	0.9991	H	-2.2736	-0.1866	-2.9466	H	0.4246	1.7081	-1.8844
C	1.5732	0.6087	-4.0883	H	-0.3730	-1.4088	-0.6410	H	-3.4775	-1.7757	-1.4791	H	0.0988	-0.1147	-0.2394
C	1.6865	1.5592	-3.0801	H	-2.0489	-1.7558	-0.2323	N	0.6267	1.1051	0.9164	H	0.9845	-0.4760	2.8257
C	0.5294	0.0983	-1.5606	C	-1.7365	1.0186	-0.6363	C	1.2228	0.6421	2.1542	H	0.5476	-2.4586	-0.9016
H	-0.0836	-1.7994	-2.3657	H	-1.8987	2.0531	-0.3284	H	2.2612	0.3589	1.9782	C	-2.1080	0.4126	1.8907
H	1.9767	0.8072	-5.0731	H	-2.6613	0.6531	-1.0901	H	0.6909	-0.2415	2.5120	H	-2.4431	-0.5980	1.6507
H	2.1807	2.5029	-3.2754	H	-0.9626	1.0172	-1.4073	H	1.1982	1.4040	2.9536	H	-2.4807	1.0850	1.1162
H	1.2589	2.0525	-1.0338	H	-2.0938	0.1982	1.3287	C	1.3494	2.2194	0.3340	H	-2.5675	0.7069	2.8514
N	-1.4719	-0.7375	3.4546	H	-0.4160	0.5417	0.9943	H	2.3839	1.9290	0.1464	[6⋯12]			
C	-1.7680	-0.7036	4.5640	C	2.4386	-0.2805	0.5236	H	1.3475	3.1157	0.9791	C	-2.1290	0.8154	1.2566
C	-2.1429	-0.6597	5.9691	H	3.3838	-0.6763	0.1591	H	0.9017	2.4882	-0.6245	C	-1.1512	-1.7828	1.4482
H	-3.2234	-0.5441	6.0648	H	1.7224	-1.0878	0.6447	C	-0.7948	1.3581	1.0407	C	-2.5691	-0.0201	2.2789
H	-1.6535	0.1821	6.4611	H	2.5677	0.2972	1.4314	H	-1.0284	2.1673	1.7549	C	-1.1995	0.3517	0.3312
H	-1.8415	-1.5824	6.4667	N	1.9079	0.6252	-0.5451	H	-1.3011	0.4505	1.3741	C	-0.7115	-0.9480	0.4263
[2⋯3]				O	1.5836	0.0965	-1.5940	[5⋯1]$^{\pi B}$				C	-2.0797	-1.3189	2.3753
O	-2.4633	1.4916	1.3185	O	1.8404	1.8158	-0.3018	C	0.5346	0.6561	-1.3039	O	1.4473	0.3133	-1.6489
C	-2.3269	1.6930	2.5052	[4a⋯8]				C	0.7769	-0.3145	1.2824	C	2.3259	0.7617	-0.6367
C	-1.0661	1.3048	3.2420	F	-1.2788	-0.9011	-0.6538	C	0.8297	-0.6825	-1.0912	H	1.8368	0.5810	0.3191
H	-0.7230	2.1175	3.8862	C	-2.1676	-0.1767	0.0118	C	0.3613	1.5093	-0.2236	H	2.5358	1.8353	-0.7351
H	-0.2874	1.0263	2.5366	F	-1.9811	1.1100	-0.2614	C	0.4824	1.0239	1.0701	H	3.2792	0.2170	-0.6631
H	-1.2837	0.4530	3.8927	F	-3.3910	-0.5336	-0.3417	C	0.9510	-1.1675	0.2022	C	1.9667	0.4922	-2.9494
C	-3.4201	2.3388	3.3255	F	-2.0047	-0.3731	1.3166	F	0.4046	1.1196	-2.5474	H	1.2238	0.1154	-3.6515
H	-3.0815	3.3170	3.6780	C	1.4271	0.4976	0.5220	F	0.0669	2.7942	-0.4283	H	2.9045	-0.0626	-3.0888

H	-3.6394	1.7399	4.2131	H	2.4032	0.8461	0.8542	F	0.3037	1.8418	2.1090	H	-2.4241	-1.9693	3.1700
H	-4.3182	2.4607	2.7241	H	0.9718	-0.1268	1.2835	F	0.8733	-0.7871	2.5273	H	-0.7724	-2.7949	1.5220
I	-0.3900	-0.0033	-0.5371	H	0.7999	1.3330	0.2311	F	1.2114	-2.4596	0.4069	H	-3.2941	0.3394	2.9989
C	1.0225	-0.9335	-1.8133	N	1.6710	-0.3505	-0.6894	F	0.9770	-1.5062	-2.1278	H	-2.5124	1.8257	1.1808
C	2.8654	-2.1368	-3.5131	O	1.9590	-1.5176	-0.4969	H	-2.9593	-0.0921	-0.3410	H	-0.8445	0.9898	-0.4678
C	0.8560	-0.8409	-3.1915	O	1.5911	0.1927	-1.7759	O	-2.2441	-0.7027	-0.1350	H	0.0105	-1.2942	-0.3019
C	2.1071	-1.6225	-1.2792	[4a···5]				H	-2.5694	-1.2326	0.6005	H	2.1567	1.5526	-3.1642
C	3.0251	-2.2231	-2.1348	C	-0.4375	1.9134	-1.3338	[5···6]^{HB}				$[N{\equiv}C^{-}\cdots C_6F_6]^{-}$			
C	1.7798	-1.4448	-4.0373	C	-1.0613	0.4591	0.9406	C	1.3015	2.6919	-2.1422	C	-0.6138	1.1958	-0.4500
H	0.0133	-0.3039	-3.6055	C	-1.2292	0.7777	-1.4348	C	2.5950	0.3012	-2.7208	C	0.7688	-1.1989	-0.4500
H	2.2357	-1.6940	-0.2079	C	0.0386	2.3241	-0.0957	C	0.9833	1.5400	-1.4347	C	0.7688	1.1958	-0.4500
H	3.8676	-2.7609	-1.7181	C	-0.2721	1.5955	1.0433	C	2.2679	2.6483	-3.1393	C	-1.3051	-0.0016	-0.4500
H	1.6459	-1.3718	-5.1096	C	-1.5436	0.0512	-0.2949	C	2.9147	1.4531	-3.4281	C	-0.6138	-1.1989	-0.4500
H	3.5814	-2.6070	-4.1748	F	-1.6758	0.3797	-2.6220	C	1.6293	0.3453	-1.7236	C	1.4601	-0.0016	-0.4500
[2···10]				F	-0.1294	2.6065	-2.4269	F	0.6776	3.8355	-1.8677	F	-1.2828	2.3547	-0.4347
I	0.0883	-1.1211	2.0555	F	0.8007	3.4118	-0.0034	F	0.0531	1.5792	-0.4814	F	-2.6432	-0.0016	-0.4347
C	0.0830	-0.0602	0.2207	F	0.1960	1.9755	2.2317	F	1.3191	-0.7604	-1.0474	F	-1.2828	-2.3578	-0.4347
C	0.0700	1.3046	-2.1955	F	-1.3360	-0.2650	2.0323	F	3.2115	-0.8455	-3.0001	F	1.4379	-2.3578	-0.4347
C	-0.3706	-0.6997	-0.9275	F	-2.2896	-1.0489	-0.3855	F	3.8389	1.4106	-4.3855	F	2.7983	-0.0016	-0.4347
C	0.5290	1.2561	0.1754	C	1.3334	-2.3335	0.9183	F	2.5716	3.7511	-3.8206	F	1.4379	2.3547	-0.4347
C	0.5199	1.9335	-1.0402	H	2.1900	-1.9315	1.4479	H	-3.0350	0.6495	3.0147	C	0.0775	-0.0016	2.6145
C	-0.3746	-0.0110	-2.1357	H	1.4089	-3.4008	0.7370	C	-2.5591	-0.2893	2.7599	N	0.0775	-0.0016	3.7835
H	-0.7156	-1.7237	-0.8860	H	0.4092	-2.1081	1.4481	C	-1.3356	-2.7026	2.1052	$[N{\equiv}C^{-}\cdots C_3N_3F_3]^{-}$			
H	0.8801	1.7487	1.0713	N	1.2353	-1.6459	-0.4084	C	-1.7845	-0.3873	1.6084	N	0.6825	-1.1821	-0.7124
H	0.8677	2.9582	-1.0798	O	1.7742	-0.5575	-0.5185	C	-2.7215	-1.3985	3.5844	C	-0.6288	1.0891	-0.6879
H	-0.7265	-0.5095	-3.0304	O	0.5882	-2.2034	-1.2754	C	-2.1105	-2.6049	3.2567	C	-0.6288	-1.0891	-0.6879
H	0.0673	1.8361	-3.1385	[4a···9]				C	-1.1727	-1.5934	1.2811	C	1.2575	0.0000	-0.6879
C	-0.2800	-3.0963	5.0621	N	-0.4240	1.4239	1.4301	H	-1.6537	0.4728	0.9650	N	0.6825	1.1821	-0.7124
O	-0.6381	-3.8157	5.8487	C	-0.5039	0.3003	1.6611	H	-3.3234	-1.3235	4.4817	N	-1.3650	0.0000	-0.7124
[2···11]				C	-0.5897	-1.1278	1.9197	H	-2.2385	-3.4678	3.8988	F	-1.2904	-2.2350	-0.7059
C	-3.2298	2.2853	-1.0141	H	-1.6081	-1.4761	1.7472	H	-0.5692	-1.6638	0.3854	C	0.0000	0.0000	2.1940
C	-1.6736	0.0853	-1.7002	H	-0.3063	-1.3412	2.9506	H	-0.8599	-3.6416	1.8501	F	-1.2904	2.2350	-0.7059
C	-3.5398	1.5464	-2.1501	H	0.0813	-1.6551	1.2406	[5···6]$^{\pi B}$				F	2.5807	0.0000	-0.7059
C	-2.1459	1.9319	-0.2157	C	0.5441	1.1691	-1.8044	C	-1.2958	0.5337	-1.7529	N	0.0000	0.0000	3.3622
C	-1.3725	0.8291	-0.5640	H	0.2803	1.8100	-0.9639	C	1.2824	-0.5171	-1.8510	$[Cl^{-}\cdots C_6H_4O_2]^{-}$			
C	-2.7594	0.4476	-2.4900	H	-0.1556	1.2988	-2.6232	C	-0.2179	1.3553	-1.8289	C	-0.1410	0.0358	0.8101
O	2.8751	-0.8382	2.0256	H	1.5771	1.3213	-2.0982	C	-1.1239	-0.8449	-1.8052	C	0.3110	-0.0673	-1.9628
C	2.5189	-2.1041	2.5465	N	0.4069	-0.2291	-1.2960	C	0.1450	-1.3705	-1.8551	C	-0.0841	1.2064	0.0518
H	1.8108	-1.9342	3.3563	O	1.3869	-0.7392	-0.7790	C	1.0658	0.8080	-1.8306	C	0.0335	-1.1865	0.1586
H	3.3959	-2.6334	2.9410	O	-0.6891	-0.7549	-1.3847	F	-2.5446	1.0495	-1.7699	C	0.2571	-1.2391	-1.2142
H	2.0428	-2.7310	1.7816	[4a···3]				F	-2.1773	-1.6949	-1.7734	C	0.1400	1.1561	-1.3217
C	3.8125	-0.9216	0.9697	O	-0.2839	1.5169	-0.2552	F	0.3444	-2.7106	-1.8151	Cl	-0.8618	0.1685	5.8684
H	4.0055	0.0941	0.6279	C	-0.0979	1.0002	0.8273	F	2.5154	-1.0187	-1.8646	H	-0.2167	2.1649	0.5398
H	3.4201	-1.5111	0.1313	C	1.2944	0.7212	1.3390	F	2.1406	1.6032	-1.8586	H	-0.0069	-2.1053	0.7320
H	-2.9930	-0.1345	-3.3730	H	1.3903	0.9812	2.3949	F	-0.4243	2.6970	-1.8196	H	0.3890	-2.1999	-1.7010
H	-1.0713	-0.7714	-1.9702	H	2.0283	1.2598	0.7439	H	-2.2711	0.9450	1.8621	H	0.1809	2.0768	-1.8941
H	-4.3853	1.8243	-2.7665	H	1.4846	-0.3518	1.2451	C	-1.2651	0.5451	1.8356	H	0.4842	-0.1079	-3.032
H	-3.8332	3.1423	-0.7418	C	-1.2437	0.5693	1.7092	C	1.3130	-0.5156	1.7791	I	-0.4853	0.0974	2.9652
H	-1.9115	2.5088	0.6684	H	-1.2047	1.1033	2.6621	C	-0.1600	1.3985	1.7893	$[N{\equiv}C^{-}\cdots C_3N_3F_3]^{-}$			
H	4.7534	-1.3741	1.3087	H	-1.1514	-0.4969	1.9281	C	-1.0699	-0.8219	1.8377	C	-0.0564	-0.8740	-0.9610
I	0.2803	0.2584	0.6288	H	-2.1925	0.7629	1.2142	C	0.2103	-1.4002	1.8002	C	0.9587	-0.9647	0.1176
												C	0.7097	-0.4880	1.3449
												C	-1.1521	0.0981	-0.7202
												C	-1.3978	0.5747	0.5079
												C	-0.5115	0.2770	1.6435
												C	1.2561	1.1002	-1.7889
												H	-1.7923	0.3036	-1.5684
												H	-2.2381	1.2251	0.7204
												H	1.4030	-0.6154	2.1679
												H	1.8480	-1.5331	-0.1226
												N	1.8753	1.9518	-2.2877
												O	-0.1379	-1.7260	-1.8352
												O	-0.7648	0.6707	2.7817
